# Rethinking Conservation and Restoration Strategies of Endangered and Key Medicinal *Clavicarpa* Plants in Yunnan‐Kweichow Plateau's Karst Areas Under Climate Change

**DOI:** 10.1002/ece3.70790

**Published:** 2025-01-14

**Authors:** Chao Luo, Baiyang He, Yulu Wu, Yuteng Xue, Huang Deng, Shanman Li, Xianghong Dong, Litang Lu

**Affiliations:** ^1^ College of Forestry Guizhou University Guiyang China; ^2^ College of Life Science Guizhou University Guiyang China; ^3^ Key Laboratory of Animal Genetics, Breeding and Reproduction in the Plateau Mountainous Region, Ministry of Education Guizhou University Guiyang China; ^4^ College of Animal Science Guizhou University Guiyang China

**Keywords:** *Clavicarpa* species, climate change, conservation status, Karst forests, Maxent model, species distributions

## Abstract

The *Clavicarpa* species, valued for their pharmaceutical, ornamental, and economic importance, exhibit notable rarity and endemism in the Karst areas of the Yunnan‐Kweichow Plateau in China. These species face significant threats from habitat loss and fragmentation, leading to a decline in biodiversity. To mitigate these threats, the Maxent algorithm was employed to analyze current and future distribution patterns, with a particular focus on the influence of climate variables in predicting potential distribution shifts and assessing extinction risks under the optimistic SSP1‐2.6 and the pessimistic SSP5‐8.5 socioeconomic scenarios. The EC‐Earth3‐Veg, MRI‐ESM2‐0, and MPI‐ESM1‐2‐HR models were utilized for conservation status assessment and project future distributions for four time periods: the present, 2030s, 2050s, and 2070s. The minimum temperature during the coldest month (Bio 6) was identified as the most critical environmental factor, influencing both habitat contraction and expansion. Our modeling indicates that regions such as South, Central, and East China, particularly areas east of the Aihui‐Tengchong line and south of the Yangtze River, exhibit the highest suitability for *Clavicarpa* species within the geographical coordinates of 18° N–45° N and 97° E–120° E. Conversely, climate change projections suggest a habitat expansion for *Impatiens claviger*, *Impatiens tubulosa*, *Impatiens pritzelii*, and *Impatiens apalophylla*, while *Impatiens guizhouensis* and *Impatiens wilsonii* face increased extinction risks. Specifically, *I. claviger*, *I. tubulosa*, and *I. apalophylla* are expected to shift northward, necessitating potential relocation to southern regions, while *I. guizhouensis* and *I. wilsonii* are projected to experience habitat losses of over 23.94% and 9.13%, respectively. Our research provides a robust scientific foundation for the conservation and sustainable utilization of these important pharmaceutical species and offers a framework for effective biodiversity management. We recommend using protected areas as a basis for the future conservation, breeding, cultivation, and utilization of *Clavicarpa* species.

## Graphical abstract

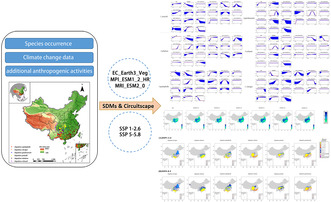

## 1 Introduction

The anticipated effects of climate change are expected to significantly impact the abundance of numerous plant and animal species in the coming century. Climate change plays a crucial role in determining the distribution of species and their habitats (Root et al. 2003). Changes in regional air temperature, vegetation, precipitation, and other climate variables due to global climate change are primary drivers of wild plant species' responses. These responses include shifts in the expansion and contraction of their ranges and alterations in population dynamics, threatening their distribution (Feng et al. 2016; Castellanos et al. 2019). Habitat loss and fragmentation are largely driven by factors such as the direct or indirect exploitation of organisms, increased human activities, land‐use changes, and overexploitation. These factors lead to the degradation and fragmentation of habitats, significantly impacting biodiversity and leading to species endangerment (Bianbaciren et al. 2009; Early and Sax 2011). Thus, it is essential to comprehensively understand the current and future distribution and biodiversity of natural habitats and to implement effective conservation measures for crucial endangered and scarce resources.

The *Clavicarpa* species is notably rare and exhibits significant endemism in China. These species are extensively used in traditional Chinese medicine in areas inhabited by ethnic minorities due to their pharmaceutical and chemical properties, treating ailments such as beriberi, fingernail inflammation, rheumatism, bruises, snakebite, and onychomycosis (Luo, Jiang, and Tang 2015; Jiang et al. 2018). In response to climate change, the species shows a preference for mesohumid areas that are moist and partly shaded, such as valleys, rivers, streams, roadside ditches, and woodland edges. Previous studies have highlighted the importance of climate in shaping the evolutionary trajectory of traits and its substantial impact on geographic distribution and natural habitats (Yu et al. 2015; Luo et al. 2022). The *Clavicarpa* species exhibit rich diversity in southwestern regions, particularly on the Qinghai–Tibet Plateau and the Yunnan‐Guizhou Plateau, where the complex terrain and variable climatic conditions provide favorable ecological niches for species diversification. Traditionally, the classification of these species has relied on morphological, palynological, and anatomical characteristics. However, the morphological features of the *Clavicarpa* species show significant variation under different environmental and climatic conditions, posing challenges to species classification. Notably, the flowers display bilateral symmetry, with a wide range of corolla colors and sizes, but this morphological diversity across different climatic zones increases the uncertainty in classification. Moreover, the stems and leaves of the *Clavicarpa* species have a semi‐succulent structure, which presents technical challenges in specimen collection and preservation, further exacerbating the difficulties in defining species boundaries. Endemic species are crucial for understanding evolutionary history and genetic diversity within and among populations. However, the specific ways in which global climate influences the present and future distribution patterns and natural habitats of important pharmaceutical and ornamental plant resources have yet to be thoroughly investigated.

Species distribution models (SDMs), which are developed using machine learning algorithms, are recognized as effective tools extensively employed across spatial and temporal scales for the distribution and conservation of natural habitats (Hastie, Tibshirani, and Friedman 2009; Brown 2014). Among various species distribution models, maximum entropy (Maxent) demonstrates superior performance in monitoring and assessing a diverse array of factors, including species invasions, habitat predictions, species reintroductions, the identification of new protected areas, and the impacts of climate change on biodiversity. Furthermore, Maxent models provide enhanced versatility in the integration of continuous and categorical data types through the inclusion of environmental variables (Elith et al. 2011; Wang et al. 2016).

Historically, research on this genus has primarily focused on morphology, taxonomy, genome evolution, and phylogenetics (Chen 2001; Fujihashi, Akiyama, and Ohba 2002; Janssens et al. 2009; Jiang et al. 2017; Luo et al. 2022). However, there has been limited exploration in previous literature regarding the effects of climate change and anthropogenic activities on species distribution and biodiversity from a broader perspective. The main objective of this study was to utilize Maxent to analyze three distinct time periods under optimistic SSP1‐2.6 and pessimistic SSP5‐8.5 socioeconomic scenarios using MPI‐ESM1‐2‐HR, EC‐Earth3‐Veg, and MRI‐ESM2‐0 models to forecast species population distribution and infer biological population richness. The main goals as follows: What is the present potential geographic range of *Clavicarpa* species? What are the primary environmental variables influencing the distribution of *Clavicarpa* species habitats in China? Where will the potential habitat distribution of *Clavicarpa* species be located under projected future climate conditions, and how will the suitable area alter under various future climate scenarios? Lastly, what proactive measures can be implemented to offer scientifically sound and efficient suggestions for the conservation of *Clavicarpa* species in light of diminishing and disappearing habitats due to climate change?

## 2 Materials and Methods

### 2.1 Study Area and Species Data

**FIGURE 1:**
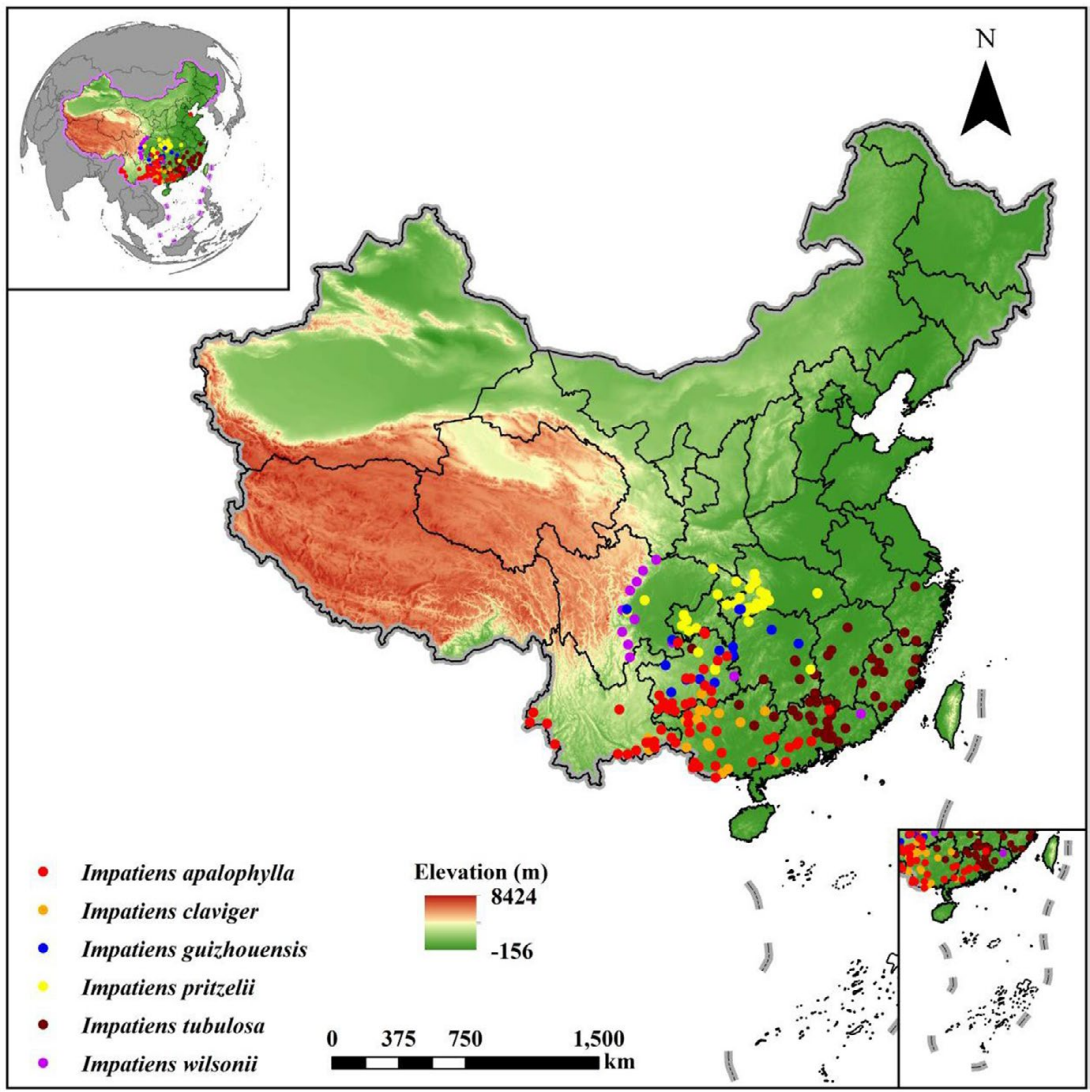
Map showing spatial distribution locations of the *Clavicarpa* species in China.

The research was conducted within the native distribution range of wild *Clavicarpa* species, spanning from 97.79° E to 120.14° E longitude and 21.55° N to 37.88° N latitude. The overlapping geographic distribution of the six *Clavicarpa* species is predominantly found in southern China, specifically in the southern region of the Yangtze River, the South China Plain, the Sichuan Basin, the Yunnan‐Guizhou Plateau and parts of the Gaoligong Mountains in Yunnan. In fact, the calibration area of our models in this project is the whole China. However, the above regions are only the main distribution areas of these six *Clavicarpa* species in China. This study specifically examined the selection of *Clavicarpa* species, including *Impatiens tubulosa*, *Impatiens apalophylla*, *Impatiens wilsonii*, *Impatiens pritzelii*, *Impatiens guizhouensis*, and *Impatiens claviger*. All species presented in this paper are considered typical representatives of rare and endemic plants found in China (Figure 1). They are indigenous to China and are extensively exhibited significant pharmaceutical and ornamental value (Ruchisansakun et al. 2015).

### 2.2 The Distribution Data of *Clavicarpa* Species

The distribution data of *Clavicarpa* species were obtained through a comprehensive review of academic literature, including articles, monographs, dissertations, and various databases such as the Chinese Virtual Herbarium (http://www.cvh.ac.cn), the Herbarium of the Institute of Botany, the National Specimen Information Infrastructure (http://www.nsii.org.cn), and the Global Biodiversity Information Facility (GBIF; https://www.gbif.org/). Records with insufficient geographical information or duplicates were omitted from the analysis. We utilized a spatial resolution of 2.5 arc‐minutes, equivalent to approximately 4.5 km^2^ at the equator (Fick and Hijmans 2017; Yang et al. 2022). To mitigate sampling bias arising from presence records, we limited occurrences to one per environmental grid cell. Additionally, we implemented an environmental subsampling method, selecting a single point from each distinct bin combination for modeling purposes (Elith et al. 2011; Lu et al. 2022). After removing erroneous and duplicated records, accurate latitude and longitude data were obtained. We collected 119 distribution records of *I. tubulosa*, 123 of *I. apalophylla*, 30 of *I. wilsonii*, 65 of *I. pritzelii*, 32 of *I. guizhouensi*s, and 53 of *I*. *claviger* , respectively (Appendix S1).

### 2.3 Environmental Data Sources

The incorporation of L_−1_ regularization in the Maxent algorithm enables experts to preselect variables during modeling, thereby mitigating the necessity to eliminate pertinent variables or preprocess covariates (Hastie, Tibshirani, and Friedman 2009; Moilanen, Wilson, and Possingham 2009; Elith et al. 2011). In accordance with previous studies, we incorporated 19 bioclimatic variables (Bio1–Bio19) alongside two nonbioclimatic variables: elevation (Elev, m) and human population density (HPD, ind/km^2^) (Gao 2020; Zhang et al. 2021) (Table S1). Owing to the absence of projected datasets for future elevation in China, elevation serves as an indicator of current environmental conditions (Leonard et al. 2017; Abdulwahab, Hammill, and Hawkins 2022), whereas HPD reflects both current and anticipated population densities (Gao 2020). The 19 bioclimatic variables were sourced from WorldClim Version 2.0, and both current (1970–2000) and future climatic conditions for the 2030s–2070s were extracted from the WorldClim database.

### 2.4 Climate Change Scenario Simulation

**FIGURE 2:**
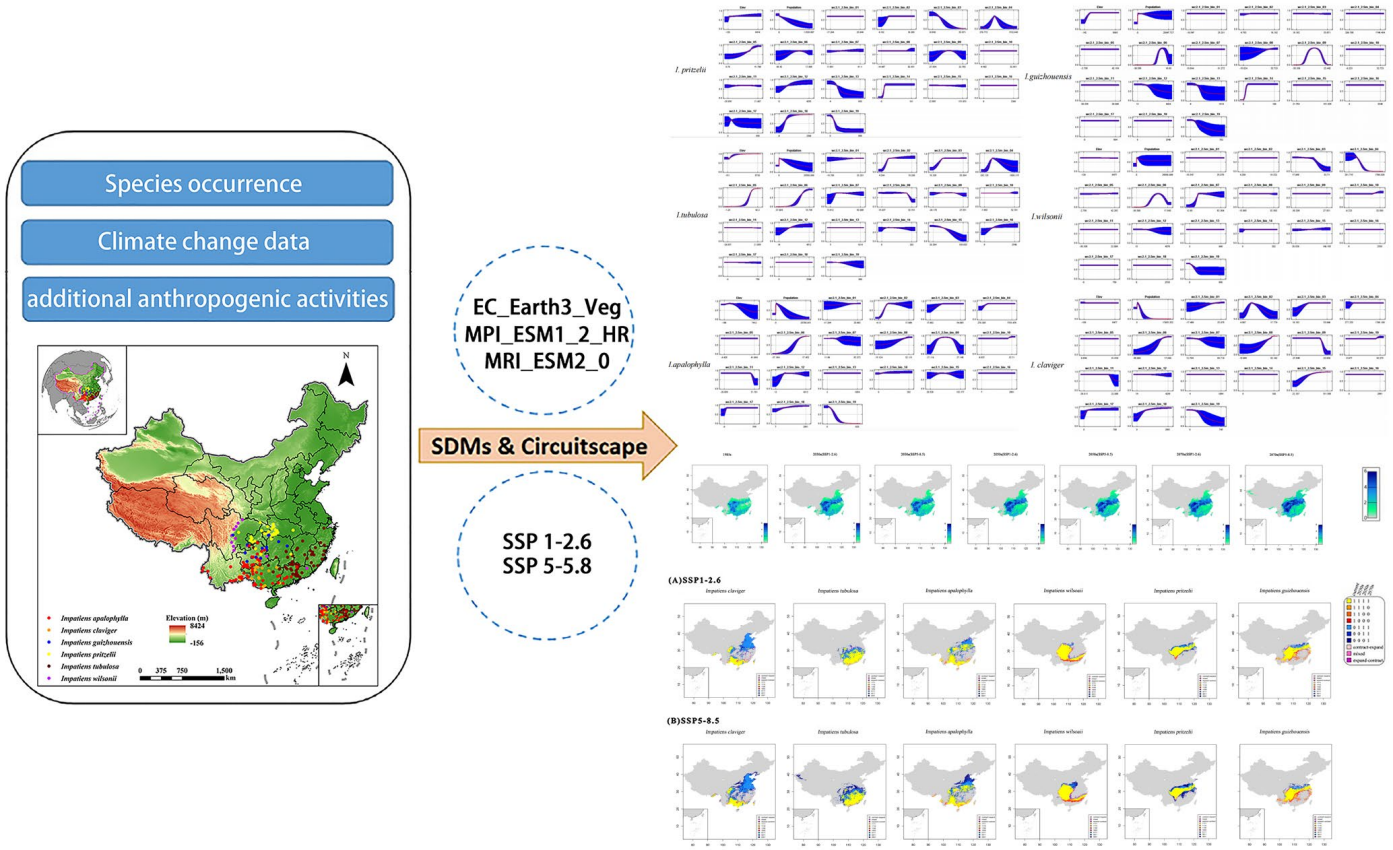
Schematic of the methodological approaches for *Clavicarpa* species.

To project changes in species distribution under future climatic conditions, we employed the SSP1‐2.6 and SSP5‐8.5 scenarios, denoting optimistic sustainable development and pessimistic fossil fuel‐driven development, respectively (Table S2). To ensure accuracy, we selected three high‐performing global climate models (MPI‐ESM1‐2‐HR, EC‐Earth3‐Veg, and MRI‐ESM2‐0) from the Coupled Model Intercomparison Project Phase 6 for projections specifically focusing on China (Hausfather 2019; Lu et al. 2022). The three GCMs were taken from the Coupled Model Intercomparison Project Phase 6 (CMIP6) and have been testified to perform well in China (Lu et al. 2022), while SSP1‐2.6 and SSP5‐8.5 simulate sustainable development (optimistic) scenario and fossil fuel‐driven development (pessimistic) scenario, respectively (Zhou et al. 2021). And the schematic of the methodological approaches for Clavicarpa species as follows (Figure 2).

### 2.5 Maxent Modeling

To develop species distribution models (SDMs) for *Clavicarpa* species, we used the ‘megaSDM’ package (version 1.1.0) in R version 4.2.1 (Kong et al. 2021; R Core Team 2022; Shipley et al. 2022). This software package integrates subsampling methods, the Maxent algorithm, and diverse statistical techniques to generate temporal visualizations depicting changes in species distribution range expansion or contraction over a designated time period (Merow, Smith, and Silander 2013). The Maxent platform provides a valuable opportunity to investigate species range shifts within the framework of climate change. It integrates recent advancements, including the generation of spatially constrained background points (BPs) and the application of environmental filtering to species occurrence records (SORs) (Shipley et al. 2022). These innovations have been demonstrated to effectively mitigate the inherent sampling bias present in species occurrence records, thereby significantly enhancing the predictive accuracy of the model (Castellanos et al. 2019). Specifically, for each species, 1000 pseudoabsence points were randomly selected and replicated. 50% of the background points were randomly distributed within a buffer surrounding the occurrence points, with the buffer radius set at twice the 95% quantiles of the minimum interpoint distance. Furthermore, we performed environmental subsampling for both the SORs and BPs. Subsequently, we employed the Maxent algorithm following established modeling protocols, utilizing default parameters whereby 80% of the occurrence data was designated for model training and the remaining 20% was allocated for validation (Phillips, Anderson, and Schapire 2013). A jackknife test was conducted to assess the significance of the variables, and response curves were utilized to depict the influence of these variables. To mitigate potential biases arising from data partitioning, we conducted 10 replicate model runs, resulting in the generation of 10 base models for each species (Bae, Murphy, and García‐Berthou 2018; Shipley et al. 2022). In our methodology, we performed a detailed adjustment of models with multiple hyperparameters. For each species, we specified the model adjustments and provided justifications for these choices. The feature classes were set as follows: for datasets with fewer than 10 records, only linear features were utilized. For species distribution models (SDMs) developed with 10 to fewer than 15 records, quadratic features were incorporated. Hinge features were included for species with 15 or more records. Additionally, for species represented by 80 or more records, Maxent incorporated product and threshold features.

### 2.6 Maxent Model Evaluation

Model accuracy was assessed using statistical measures such as the receiver operating characteristic curve (AUC) (Allouche, Tsoar, and Kadmon 2006). The AUC value is plotted on the ROC curve, with the false‐positive rate on the *x*‐axis and the true‐positive rate on the *y*‐axis. The ROC curve plots the relationship between sensitivity and (1—specificity) at all possible thresholds between 0 and 1. Here, sensitivity represents the proportion of correctly predicted presence cases, while specificity represents the proportion of correctly predicted absence cases. If the curve lies above the no‐discrimination diagonal line, that is, AUC > 0.5, it indicates that the model's ability to discriminate is better than random. In this study, an ensemble modeling approach was adopted, utilizing base models with AUC values categorized as fair (0.7–0.8), good (0.8–0.9), and excellent (0.9–1). Habitat suitability was subsequently converted into binary (presence/absence) projections by applying the relevant thresholds for maximum test sensitivity and specificity (Shipley et al. 2022).

Using the maximum entropy algorithm (Maxent 3.3.3e; Phillips, Anderson, and Schapire 2006), we initially employed SDMs based only on the less‐correlated climatic predictors (settings used: 10 bootstrap replicates, auto features, random test percentage = 25, regularization multiplier = 1, maximum iterations = 500, and convergence threshold = 0.0001) (Rathore, Roy, and Karnatak 2022). Through the application of species‐specific thresholds, we delineated grid cells predicted as “presence” across all model iterations into a composite climatic suitability range (CLIMATE) for each species, thereby encapsulating the potential habitat range. Considering the inherent limitations of climatic data, characterized by a relatively coarse effective spatial resolution and a sparse sample density (Franklin 2009), which are predominantly influenced by the density of the station network and the interpolation methodologies employed (Hijmans et al. 2005; Cord et al. 2014), We assembled species richness maps at a spatial resolution of 1 km^2^. For comparative analysis, we preserved the summed suitability surfaces devoid of threshold application, eschewing any further modifications (Calabrese et al. 2014). The createTimeMaps function combines the set of binary distribution maps generated across all time steps into a single raster file displaying the regions vacated and migrated to during each time period. The value of each raster pixel is given as code describing presence (1) and absence (0) for each time step (Shipley et al. 2022). The resulting six binary species distribution maps were then aggregated to generate species richness maps, which depict the spatial and temporal patterns and trends of species richness. Additionally, future projections were conducted using two other highly effective general circulation models (GCMs), namely MRI‐ESM2‐0 and EC‐Earth3‐Veg, across China for comparative purposes. For more details on the modeling process, please refer to relevant literature or the “megaSDM” package (Shipley et al. 2022). All statistical analyses were conducted using R software (Baddeley and Turner 2005; Carver et al. 2021).

## 3 Results

### 3.1 Model Performance

**FIGURE 3:**
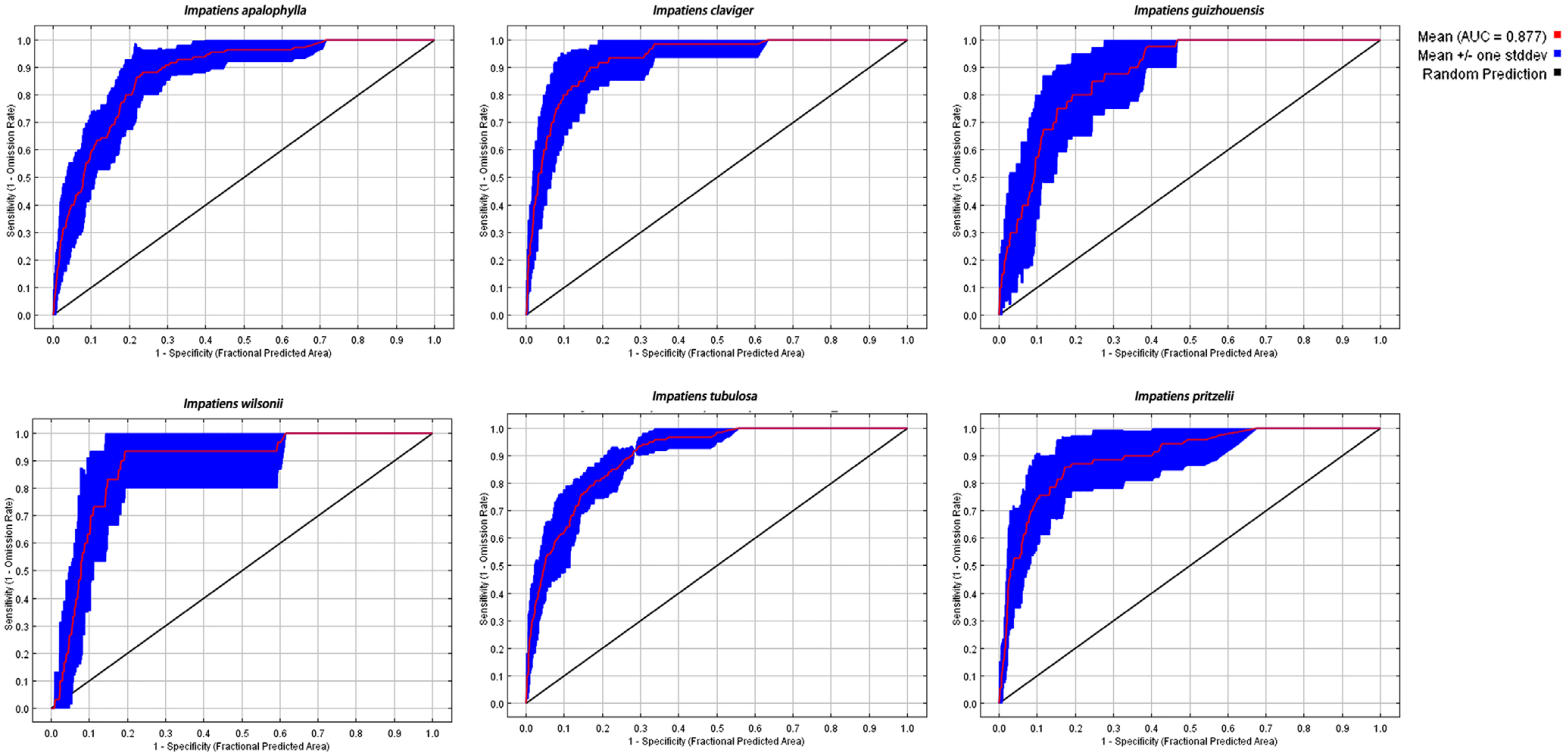
Mean model performance or generalization ability (*n* = 10, i.e., 10 repetitions) indicating by the area under the receiver operating characteristic curve (AUC). SD, standard deviation.

The “megaSDM” approach demonstrated its efficacy by producing a Maxent model for *I*. *claviger* with an average testing AUC of 0.90 ± 0.02, exceeding the threshold value of 0.50. Additionally, the AUC values for the training sets of *I. tubulosa*, *I. apalophylla*, *I. wilsonii*, *I. pritzelii*, and *I. guizhouensis* ranged from 0.8 to 0.9, underscoring the precision and reliability of our ensemble model in forecasting potential distributions (Table S3; Figure 3). These results affirm the Maxent model's capability in predicting potential distributions as per our research criteria.

### 3.2 Variable Importance

Prior studies have utilized percent contribution and jackknife analysis of the area under the curve (AUC) to determine the significance of variables (Kumar and Stohlgren 2009). As a result, variables with a permutation importance value > 10% were identified as the primary environmental factors influencing the distribution of *Clavicarpa* species. Notably, precipitation during the warmest season was identified as the primary environmental factor impacting the distribution of *I. clavager* (Table S4). The cumulative permutation importance of this factor reached a significant 57.4%. The study found that the environmental suitability of *I. claviger* was positively correlated with precipitation during the warmest season, but negatively correlated with both the annual average temperature (Bio1) and the difference in annual precipitation (Bio12). For *I. guizhouensis*, precipitation during the driest month (34%) and population size (31.7%) were the main influences, together accounting for 65.7% of the overall impact. The suitability curve for *I. guizhouensis* displayed a unimodal distribution for both variables.

**FIGURE 4:**
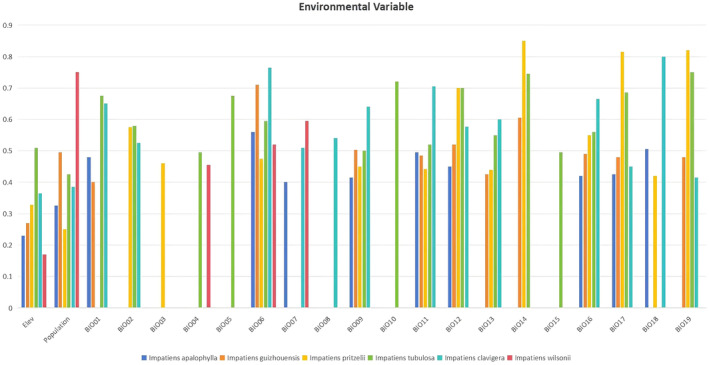
Mean variables importance relating to spatial distribution of the *Clavicarpa* species determinate by jackknife test.

*Impatiens tubulosa* was primarily influenced by precipitation during the driest month (20.4%), the coldest season (25.5%), and population size (10.5%), with an optimal cumulative influence exceeding 66.4%. The distribution of *I. apalophylla* was mainly driven by annual precipitation (22.6%) and population size (23.1%). For *I. wilsonii*, temperature seasonality (24.1%), annual precipitation (12.2%), and population size (24.5%) were the key factors, with the suitability curve demonstrating a unimodal relationship with these factors. Among the 19 bioclimatic variables, Bio6, Bio11, Bio14, Bio18, and Bio19 showed strong correlations with species distribution constraints in both training and test datasets. Conversely, isothermality, Bio8, and elevation had weaker impacts. Bio6 and Bio18 provided crucial information for identifying species presence or absence, particularly influencing habitat selection for *I. guizhouensis*, *I. apalophylla*, *I. wilsonii*, and *I. claviger* (Table S4; Figure 4).

### 3.3 Response Curves

Permutation importance offered a more accurate ranking compared to contribution percentage (Phillips et al. 2009). Response curve analysis indicated that temperature seasonality (Bio4), Bio6, and Bio7 were the primary influences for *I. wilsonii*, with Bio6 being the most crucial. For *I. apalophylla*, Bio6, Bio12, and Bio19 were the top factors, with the lowest temperature of the coldest month contributing most significantly to the SDM. *I. pritzelii*'s response curves highlighted Bio14, temperature seasonality (Bio4), isothermality (Bio3), and Bio19 as key variables (Table 1). Furthermore, it is apparent that the geographic distributions of *I. wilsonii*, *I. apalophylla*, and *I. guizhouensis* are predominantly influenced by population variables. Specifically, their distributions are delineated within the specified ranges of 0–26,560 ind/km^2^, 0–25,780 ind/km^2^, and 0–20,947 ind/km^2^, respectively. The transition to autumn occurred upon surpassing the respective values. The presence of *I. tubulosa*, *I. apalophylla*, *I. wilsonii*, *I. guizhouensis*, and *I*. *claviger* was primarily influenced by Bio 6 (Table S5; Table 1). However, the suitability of the habitat for *Clavicarpa* species gradually improved as the Bio 6 increased within the range of −38.09°C to 17.84°C, and remained constant thereafter.

**TABLE 1:** Detail information about the mean response curves of top four bioclimate variables chosen for MaxEnt modeling.

Species	Brief descriptions
*I*. *claviger*	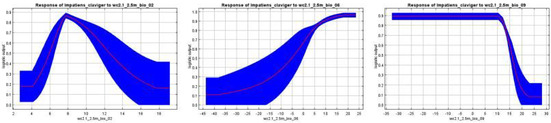
*I. guizhouensis*	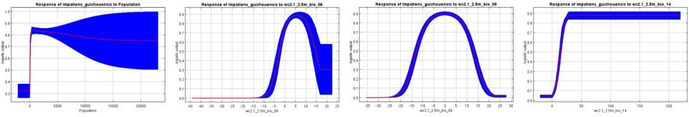
*I. tubulosa*	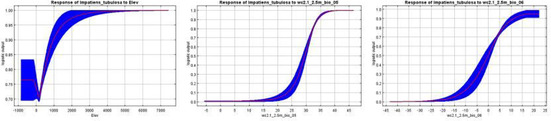
*I. apalophylla*	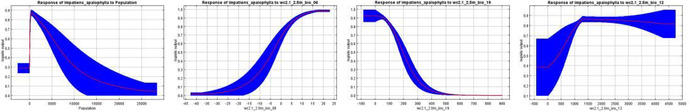
*I. wilsonii*	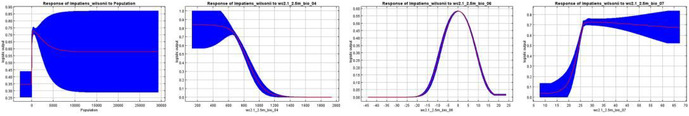
*I. pritzelii*	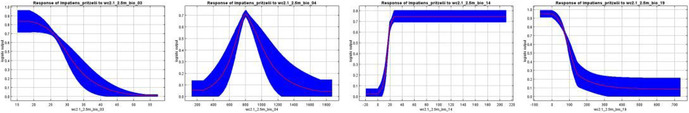

### 3.4 Potential Distribution Under Climate Scenarios

Models projected varying range shifts for *Clavicarpa* species under different Shared Socioeconomic Pathways (SSP) scenarios and time periods. *I. tubulosa*, *I. apalophylla*, *I. pritzelii*, and *I*. *claviger* exhibited range expansions with higher SSP severity, while *I. wilsonii* and *I. guizhouensis* experienced range losses. The global climate model MPI‐ESM1‐2‐HR was selected for further analysis, with additional results from other GCMs available in the Appendix S2. Substantial increases in the distribution range of *I. tubulosa*, *I. apalophylla*, and *I*. *claviger* are expected, with expansions from 38.61% to 291.90%. *Clavicarpa* species primarily inhabit roadside ditches, valleys, and streamsides between 18° N–45° N and 97° E–120° E, favoring wetlands or high moisture areas, though they also adapt to drier habitats. Potential habitat expansion is anticipated in the southern Yellow River and northern Yangtze River regions, especially within the North China Plain, Sichuan Basin, and Yangtze River Delta. *I. guizhouensis* and *I. pritzelii* are projected to have minor, inconsistent alterations, while *I. wilsonii*'s range may contract marginally, with losses from 1.06% to 12.60%. Distribution centroid shifts are expected southward for *I. pritzelii* and northward for other species by the 2070s (Table 2).

**TABLE 2:** Changes in distribution centroids and suitable grid numbers of the six species across different scenarios and time periods compared with those in the current period. Current distribution centroids (longitude, latitude).

Species	Scenarios	Time	Distribution centroid		Change in suitable grid number (%)
		Periods	Longitude	Latitude	
*I. apalophylla*	SSP1‐2.6	2030s	110.71	28.09	0.66
		2050s	110.63	28.36	−0.13
		2070s	110.97	28.58	−0.04
	SSP5‐8.5	2030s	110.83	28.06	0.56
		2050s	111.29	28.62	0.12
		2070s	111.82	28.94	0.20
*I*. *claviger*	SSP1‐2.6	2030s	111.61	28.61	141.00
		2050s	111.86	29.17	−3.90
		2070s	112.25	29.22	6.88
	SSP5‐8.5	2030s	111.99	28.92	147.26
		2050s	112.48	29.59	24.90
		2070s	112.67	29.58	22.76
*I. guizhouensis*	SSP1‐2.6	2030s	111.13	28.92	26.18
		2050s	110.62	29.36	−10.02
		2070s	110.45	29.40	−3.99
	SSP5‐8.5	2030s	110.50	28.84	20.95
		2050s	111.17	29.55	0.16
		2070s	111.24	29.75	−23.94
*I. pritzelii*	SSP1‐2.6	2030s	111.85	30.52	21.52
		2050s	111.91	30.42	9.24
		2070s	112.12	30.61	7.43
	SSP5‐8.5	2030s	111.46	30.12	18.85
		2050s	112.10	30.30	20.56
		2070s	112.35	30.10	27.32
*I. tubulosa*	SSP1‐2.6	2030s	112.70	26.83	40.17
		2050s	112.86	26.71	13.16
		2070s	112.95	26.88	0.40
	SSP5‐8.5	2030s	112.93	26.69	32.13
		2050s	112.42	26.91	15.97
		2070s	111.79	27.61	27.81
*I. wilsonii*	SSP1‐2.6	2030s	109.34	29.12	4.94
		2050s	109.24	29.09	−3.06
		2070s	108.99	29.28	−9.13
	SSP5‐8.5	2030s	109.27	29.01	2.28
		2050s	109.50	29.53	9.78
		2070s	109.11	30.01	−8.28

### 3.5 Centroid Changes in Potential Distribution

**FIGURE 5:**
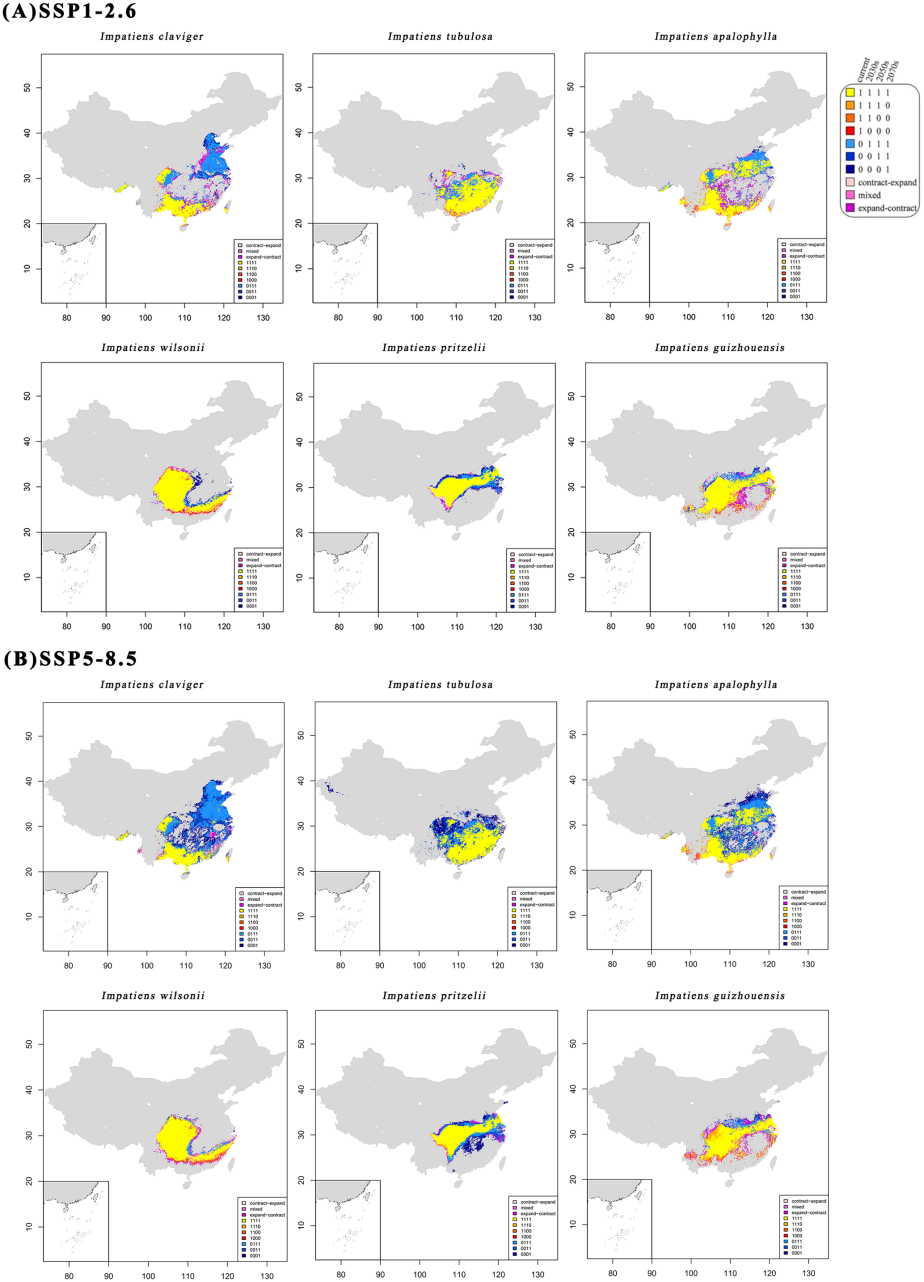
Maps depicting habitat‐suitability's step‐wise expansion and/or contraction of the six species across the global climate models (MPI‐ESM1‐2‐HR), scenarios (i.e., SSP126 and SSP585), and time periods (i.e., 1985, 2030, 2050 and 2070). SSP is the abbreviation of shared socioeconomic pathways, while “1” and “0” in the legend indicate presence and absence of one species, respectively; “1001,” “1011,” and “1101” indicate the “contract‐expand” group; “0101” and “1010” indicate the “mixed” group; “0100,” “0010,” and “0110” indicate the “expand‐contract” group.

The habitats of *Clavicarpa* species remained consistently suitable, primarily in specific regions. Under the SSP1‐2.6 scenario, the habitats of *I. tubulosa*, *I. apalophylla*, and *I*. *claviger* maintain continuous suitability expansion in specific regions. By the 2070s, significant habitat expansions for *I. apalophylla* and *I*. *claviger* are observed in the Central North China Plain, Sichuan Basin, and the Yangtze River Delta. *I. tubulosa* is expected to primarily expand its habitat in the Sichuan Basin and along the Yangtze River. Conversely, *I. wilsonii* and *I. guizhouensis* experience significant habitat degradation in the Lingnan region, the Yunnan‐Guizhou Plateau, and Central China. *I. pritzelii* is not significantly affected by the SSP1‐2.6 scenario and continues to expand along the Yangtze River region (Figure 5).

Under the SSP5‐8.5 scenario, both species detect scattered temporary fluctuations along the Central and Yangtze River coasts. The consistently suitable habitats for *I. pritzelii* and *I. guizhouensis* are located in the Karst areas of the Guizhou Plateau, along the Yangtze River, and in the Yangtze River Delta. In contrast, by the 2070s, *I. tubulosa*, *I. apalophylla*, and *I*. *claviger* are projected to expand northeastward. *I. guizhouensis* experiences scattered losses along its range boundaries under both scenarios (Table 2). The consistently favorable habitats for *I. wilsonii* are primarily situated in the Sichuan Basin, Guizhou Karst Region, Chongqing, and the Lingnan region, with expansion toward the southern regions under severe SSP scenarios. Changes from the present to the future for *I. wilsonii* and *I. guizhouensis* are independent of their current elevation and suitable area extent, particularly under the specified scenarios.

### 3.6 Current and Future Species Richness Areas

**FIGURE 6:**
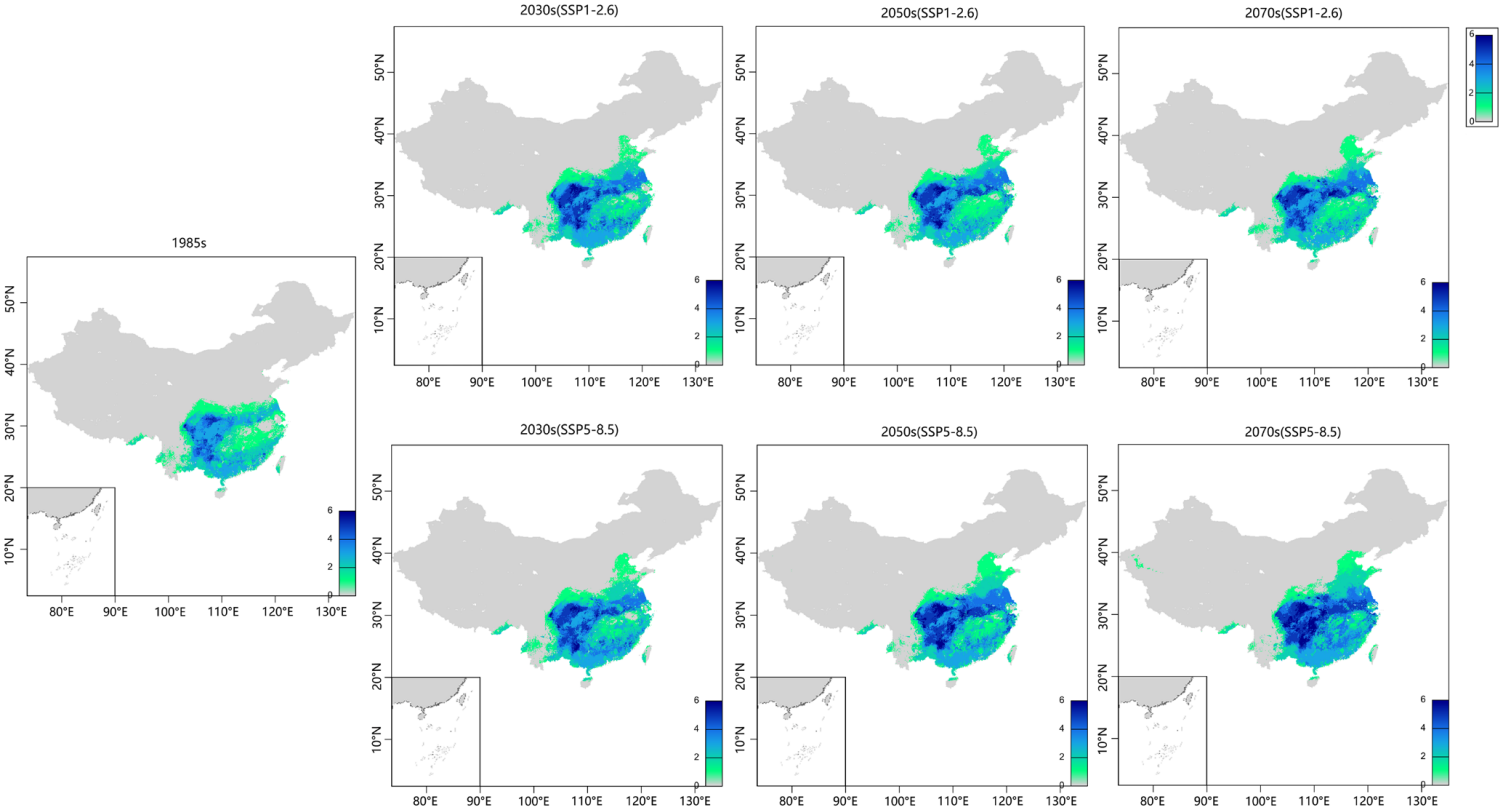
Species richness maps cumulating the six species distributions forecasted under SSP1‐2.6 and SSP5‐8.5 scenarios and for current, the 2030s, 2050s, and 2070s four time periods.

Using the MPI‐ESM1‐2‐HR model, the potential global geographic range of *Clavicarpa* species in the 2030s–2050s is shown in Figure 6. Appendix S2 provides results from other models. An overall increase in suitable habitat area is expected under both scenarios compared to current conditions. *Clavicarpa* species distributions overlap significantly in southern China, especially along the Yangtze River, South China Plain, Yunnan‐Guizhou Plateau, Sichuan Basin, and Gaoligong Mountains (Figure 6). Under the SSP1‐2.6 scenario, significant overlap is expected in southern China during the 2030s and 2050s, extending to North China, including Hunan, Hubei, and parts of Shandong. Increased species richness is projected in the Yangtze River basin, North China Plain, and South China hills, particularly along the Yangtze River with high rainfall, by the 2070s. The SSP5‐8.5 scenario projects enrichment in southwestern, central, and eastern China. By the 2070s, the Sichuan Basin, Yunnan‐Guizhou Plateau Karst region, and Yangtze River Basin are expected to have the largest expanses of suitable habitats, concentrated near ports and rivers. The Pearl River Basin, Guangxi Karst area, Lingnan area, and southern coast will have moderately suitable habitats. Increased greenhouse gas concentrations may transform non‐habitats into habitats, connecting existing habitat fragments in the future.

### 3.7 Extinction Risk and Migration Rate

This study found that increasing greenhouse gas concentrations (GHG) enhanced the extinction risk and migration rate for some *Clavicarpa* species. *I*. *claviger* , *I. tubulosa*, and *I. wilsonii* demonstrated extinction risks above 0.85% in the current scenario. However, under SSP5‐8.5, the risk for *I. guizhouensis* and *I. pritzelii* fell below 0.35%, possibly due to the increase in potential habitat area. The risk of extinction for these species aligns with their high sensitivity to changing temperature and precipitation patterns, especially during the driest and warmest months. Additionally, the findings indicate a slower migration rate for *I. wilsonii*, *I. guizhouensis*, and *I. pritzelii* due to the limited habitat expansion under high GHG scenarios. Conversely, the rapid habitat expansion for *I. tubulosa*, *I. apalophylla*, and *I*. *claviger* suggests higher migration rates, potentially reducing their extinction risk. The study emphasizes the need for conservation efforts focusing on the species with lower migration rates to ensure their survival under changing climatic conditions.

## 4 Discussion

### 4.1 Environmental Variables' Influence on Geographical Distributions

The rapid response of plant distribution and habitat to global climate change is well‐documented in the literature. However, previous studies on species distribution modeling (SDM) have predominantly concentrated on the selection of climate variables for modeling and forecasting purposes. It is crucial to acknowledge that anthropogenic influences, bioclimatic variables, and other factors play a substantial role in the observed synergistic effects (Royle et al. 2012; Yang et al. 2013). Our study utilized contributions analysis and jackknife of regularized training gain to identify significant variables impacting the trend of *Clavicarpa* species. Our findings suggest that bioclimatic variables, particularly Bio 6, Bio 11, Bio 17, Bio 18, and Bio 19, are pivotal in shaping the geographic distribution of *Clavicarpa* species. These variables play a crucial role in determining potential species ranges and influencing geographic distributions by affecting thermal and precipitation conditions. Although there are differences in bioclimatic variables among *Clavicarpa* species, their responses to Bio 6 were found to be consistent. This consistency is demonstrated by the species response curves, which suggest that areas with Bio 6 are likely to be climatically suitable for these species under two socioeconomic pathways. This forecast indicates a potential range expansion of these species towards northern regions, where they are currently absent, in response to an expected increase in mean temperature of 2.0°C–5.0°C. The suitability of habitats for *I. wilsonii* and *I. guizhouensis* significantly decreased when temperatures exceeded 33°C, indicating potential habitat loss for these species as maximum summer temperatures continue to rise. However, it is possible that these species may benefit from global warming in certain peripheral areas of their current ranges.

From an ecological standpoint, factors including niche differentiation, species adaptability, interspecific competition, habitat alterations, population dynamics, and genetic diversity may collectively influence the differential responses of *Clavicarpa* species to climate change. For instance, *I. pritzelii* may be adapted to more humid environments, whereas *I*. *claviger* demonstrates a higher tolerance to drought and elevated temperature conditions. Climate change has the potential to modify these environmental parameters, consequently impacting the distribution and survival strategies of these species. Furthermore, the genetic composition and physiological mechanisms inherent to various species significantly influence their capacity to respond to climate change. For example, *I. guizhouensis* and *I. wilsonii* may depend on particular temperature and precipitation regimes, and alterations in these climatic conditions could impede their adaptive capabilities. In contrast, other species may exhibit enhanced resilience in adjusting to the environmental transformations prompted by climate change.

Factors such as topography and human activities can independently influence species distribution. Human activities, such as population density and hydroelectric projects, have the potential to influence species distributions, as evidenced by research conducted (Vaughan and Ormerod 2005; Liu et al. 2021). Our study reveals that the presence of *I. apalophylla*, *I. wilsonii*, and *I. guizhouensis* has been notably affected by human activities, including the construction of urban areas, expansion of infrastructure, and habitat destruction. These anthropogenic actions have significantly altered the distribution and availability of suitable habitats for these species (Bellard et al. 2012). Various anthropogenic dispersal mechanisms, such as plantation planting, bee pollination, the establishment of animal and plant corridors, and the manipulation of microclimates, have been instrumental in facilitating the transmission of these species (Tan et al. 2023). While elevation is generally considered a key factor in determining habitat suitability for species, it did not significantly impact our models, except for its crucial role in determining the habitat suitability for *I. tubulosa* in our study. It is anticipated that in the future, several high‐elevation regions such as parts of the Yun‐Gui Plateau and Southeast Hilly region will exhibit favorable conditions for the habitat suitability of this particular species. Therefore, especially in the case of soil type, insolation, solar radiation, land use, plant morphology or photoperiod, which are affected by habitat transformations or pollution changes, all have different effects on the distributions of these *Clavicarpa* species.

### 4.2 Model Limitations and Effects on Potential Distribution

The reliability of our results hinges on the authenticity of the data and the integrity of the model. Unlike models developed for individual species in previous studies, the Maxent model can achieve enhanced outcomes by mitigating overfitting of training data through regularization (Brun et al. 2020). Recent research has consistently shown that Maxent models outperform other models, even with small sample sizes (Zhang et al. 2022). Furthermore, the inclusion of existing data points to generate artificial missing points during modeling limits the scope of predictions for species distribution (Khoury et al. 2019). In the absence of data regarding the presence of living specimens, a disparity between in situ and ex situ conservation efforts may arise (Lu et al. 2022). Event records sourced from online databases predominantly focus on easily accessible locations such as roads and densely populated areas (Kramer‐Schadt et al. 2013; O'Neill et al. 2017).

Consequently, populations in diverse geographical regions may display significant differences in their ability to adapt to specific environmental stressors and respond to meteorological conditions due to evolutionary processes (Austin and Van Niel 2011; Zhang et al. 2019). In addition, our model considers only two nonclimatic variables, altitude and population density, while a multitude of other factors, including the role of bees or birds in plant pollination and the effects of hydrology or wind on the dispersal of plant pollen, play a significant role in determining species distribution (Sun et al. 2020). The potential impact of global climate change on bee populations and nectar collection patterns, as well as on the diversity of plant rhizobia and endophytes, which are nonclimatic factors essential for the functioning of *Clavicarpa* species, is still uncertain (Hu, Guo, and Jin 2019).

In the context of climate change, spatial and temporal mismatches between plants and their pollinators may arise, potentially exerting a significant impact on plant dispersal and reproductive success. Climate change can lead to a desynchronization of phenological events between plants and their pollinators. For instance, rising temperatures may cause an advancement in plant flowering times, whereas the activity periods of pollinators may not adjust correspondingly. This misalignment can result in an inadequate presence of pollinators during the crucial flowering period. Secondly, climate change has the potential to induce spatial shifts in the distributions of both plants and their pollinators. Given that plants and pollinators exhibit differential responses to changing climatic conditions, such shifts may lead to a divergence in their historically overlapping ranges, thereby exacerbating pollination mismatches. These mismatches can adversely impact plant reproductive success, subsequently affecting population dynamics and dispersal capabilities. In the event of pollination failure or reduced efficiency, plants may produce an insufficient quantity of seeds, thereby constraining their ability to disperse into novel environments. Furthermore, a decrease or instability in pollinator populations may compromise plant genetic diversity, thereby diminishing their capacity to adapt to environmental changes. Consequently, the spatiotemporal mismatches between plants and their pollinators constitute a significant concern in the context of climate change, especially for plant species dependent on specific pollinators, which may encounter heightened challenges to survival and reproduction (Zhang et al. 2016). Ultimately, climate impacts are the principal drivers of species population distributions, habitat modifications, and population abundance.

SDMs provide critical insights into how climate change might alter habitat suitability for various species by integrating climatic variables with species occurrence data, thus forecasting potential shifts in species distributions and informing conservation strategies and policymaking. However, these models face significant limitations when directly assessing extinction threats due to their inability to fully account for dynamic ecological processes that can influence population sizes and survival rates. For instance, species' dispersal capabilities play a pivotal role in their capacity to colonize new habitats or retreat to more suitable environments as climatic conditions evolve, yet species with limited dispersal abilities might not track their shifting climate envelopes, potentially leading to local extinctions even in areas predicted as suitable by SDMs. Additionally, the models often overlook the potential impact of invasive species, which can invade, compete with, or predate upon native species, thereby exacerbating extinction risks. Stochastic ecological events, such as extreme weather events, fires, or disease outbreaks, can drastically impact species populations in ways that SDMs are ill‐equipped to predict. Furthermore, resource competition, a critical factor influencing species distribution and survival, is typically not accounted for in SDMs, as these models do not simulate the competitive dynamics for limited resources. To address these limitations, future research could incorporate dispersal dynamics into SDMs, considering species‐specific movement patterns and barriers to dispersal, integrate biotic interactions such as competition, predation, and facilitation to modulate species responses to climate change, model stochastic events by incorporating probabilistic elements that simulate the likelihood and impact of extreme events, and enhance model complexity to better represent the multifaceted nature of ecological processes, possibly through the use of agent‐based models or network analysis.

### 4.3 Current and Future Ranges of *Clavicarpa* Species

The results of our study suggest that climate variables have a significant impact on the geographical distribution of *Clavicarpa* species. Specifically, temperature variables are identified as key factors in determining distribution patterns. Our research highlights South China as a region with ample suitable habitats for all *Clavicarpa* species. Furthermore, we predict the presence of shared suitable habitats in the Sichuan Basin, as well as in South, Central, and East China. Moreover, it is anticipated that by the end of the century, certain species will experience range expansions in the North China Plain and Shandong Peninsula. Notably, regions situated west of the Aihui‐Tengchong Line, north of the Yangtze River, and three northeastern provinces within China were found to be unsuitable for the *Clavicarpa* species. The distribution of present and potential suitable habitats for the *Clavicarpa* species was found to be distinctly separated along the Aihui‐Tengchong Line and south of the Yangtze River, indicating a significant geographical divide. The observed phenomenon may be ascribed to variations in environmental and anthropogenic factors between the western and eastern regions. Specifically, the eastern region, characterized by lower elevation, higher precipitation, and elevated temperatures, provides more favorable conditions for the proliferation of the *Clavicarpa* species.

Additionally, due to differences in species‐specific susceptibility to global environmental changes, the *Clavicarpa* species exhibit varying degrees and directions of range expansions or contractions (Barbet‐Massin et al. 2012). It is predicted that in the face of future global changes, significant range expansions will likely occur for *I. claviger* , *I. tubulosa*, and *I. apalophylla*, while *I. wilsonii*, *I. pritzelii*, and *I. guizhouensis* are expected to experience minimal changes in range. Nevertheless, all three species, including *I. claviger* , *I. tubulosa*, and *I. apalophylla*, are projected to shift northward. On the contrary, the anticipated impacts of global changes are expected to diminish the availability of suitable habitats for *I. pritzelii*, potentially prompting their migration to southern regions. Our findings indicate a significant decrease in suitable niche space for *I. wilsonii* and *I. guizhouensis*, indicating a global reduction in the range of these species across all future time periods in response to anticipated global climate change. Therefore, it is imperative to focus on the eastern region to implement protective measures aimed at bolstering genetic diversity and alleviating the level of endangerment.

### 4.4 Conservation and Protection of Suitable Habitat for *Clavicarpa* Species

In contrast to current climate conditions, the projected future climate scenario is expected to lead to a notable decrease in the distribution range and suitable habitat of *I. wilsonii* and *I. guizhouensis*, posing a significant threat to their survival. Specifically, under the RCP 2.6 scenario, it is anticipated that approximately 63% of the habitat loss experienced by both species will have a considerable adverse impact, potentially endangering their presence in China. Consequently, these results suggest that *I. guizhouensis* is vulnerable, while *I. wilsonii* is at risk of extinction based on our projected scenarios. Moreover, our research demonstrates that the reduction in appropriate habitats for *I. apalophylla* and *I. claviger* primarily occurred on the outskirts of their distribution ranges, decreasing gradually from the outermost areas towards the central regions. This trend is especially notable in the boreal region, highlighting the critical importance of creating transboundary conservation reserves for biodiversity (Yang et al. 2013). These reserves should be strategically positioned according to existing distribution ranges and optimal dispersal routes, with the goal of preserving suitable habitats and promoting connectivity.

The genetic diversity of a population is a key determinant in the formulation of conservation strategies, as it influences the ability of a species to adapt and evolve in response to environmental changes. Factors such as environmental conditions and species characteristics also play a role in shaping genetic diversity (Bibee et al. 2011; Peng et al. 2022). Given the valuable pharmaceutical and ornamental properties of the *Clavicarpa* species, it is essential to promptly develop effective conservation strategies. To ensure the continued survival of the *Clavicarpa* species, it is crucial to enact proactive conservation measures that address the adverse impacts of global climate change (Decamps et al. 2021). Assisted colonization or translocation, a conservation strategy involving the relocation of species to habitats expected to sustain their long‐term viability under future climate conditions, presents itself as a viable approach.

Therefore, we recommend the implementation of the following conservation actions to mitigate potential future effects of climate change on the *Clavicarpa* species. Preserving the pollen and seed germplasm resources of vulnerable populations in diverse geographical areas, such as the karst regions of Guizhou, Guangxi, and Guangdong, as well as the Poyang Lake, Chaohu, and other Jiangnan waters, and the mountainous areas in the Yunnan‐Guizhou Plateau, is essential. To enhance the populations of *I. wilsonii* and *I. guizhouensis*, conduct offspring experiments, establish genetic resource protection areas, and safeguard genetic diversity, the introduction of *I. guizhouensis* to potentially suitable habitats through assisted migration is recommended. This methodology entails relocating resilient populations from unsuitable environments to more suitable habitats, encompassing various regions such as the Guizhou plateau, specific low‐altitude areas in Anhui province and Hubei province, as well as regions with abundant water sources along the southern Yangtze River. It is suggested to introduce *I. wilsonii* into the identified potentially suitable habitats, which include all karst areas in Guizhou, Chongqing, Hubei province, Hunan province, and the eastern portion of the Sichuan Basin, in order to enhance its distribution. This initiative seeks to facilitate gene flow among migrant populations in order to enhance the adaptive capabilities of native populations in anticipation of forthcoming climate change (Ghiani et al. 2012). Simultaneously, it is crucial to improve habitat connectivity within appropriate distribution zones and implement rigorous management and protection strategies for neighboring protected areas.

## 5 Conclusions

The Maxent modeling approach was used to predict the potential distribution of *Clavicarpa* species under SSP1‐2.6 and SSP5‐8.5 climate change scenarios, considering both current and future climate conditions. The findings indicate that habitat characteristics are primarily influenced by air temperature and human activities. Specifically, the minimum temperature in the coldest month (Bio 6) plays a crucial role in determining changes in the potential and overlapping ranges of these species. Additionally, the projections show that the potential distribution and areas of overlap for *Clavicarpa* species are concentrated in regions east of the Aihui‐Tengchong line and south of the Yellow River, including the Sichuan Basin, Guizhou Karst Plateau, and adjacent areas. Climate change has resulted in habitat expansion for *I. claviger* , *I. tubulosa*, *I. pritzelii*, and *I. apalophylla*, while *I. guizhouensis* and *I. wilsonii* are at a higher risk of extinction based on our scenarios. Some species may shift northward, with *I. pritzelii* potentially moving toward southern regions. In the studied area, *I. guizhouensis* and *I. wilsonii* are projected to decline by over 23.94% and 9.13%, respectively. This study is the first to assess the immediate range dynamics of *Clavicarpa* species in China. It not only provides a scientific basis for the conservation and sustainable use of these important pharmaceutical species but also holds practical significance in establishing an effective conservation and biodiversity management framework.

## Author Contributions

**Chao Luo:** conceptualization (equal), data curation (equal), formal analysis (equal), funding acquisition (equal), investigation (equal), methodology (equal), project administration (equal), resources (lead), software (lead), supervision (lead), validation (lead), visualization (lead), writing – original draft (lead), writing – review and editing (lead). **Baiyang He:** conceptualization (equal), data curation (equal), formal analysis (equal), resources (equal), software (equal). **Yulu Wu:** data curation (equal), investigation (equal). **Yuteng Xue:** resources (equal), software (equal), supervision (equal). **Huang Deng:** resources (equal), software (equal). **Shanman Li:** resources (equal), software (equal). **Xianghong Dong:** software (lead), writing – review and editing (supporting). **Litang Lu:** conceptualization (supporting), data curation (supporting), writing – review and editing (supporting).

## Conflicts of Interest

The authors declare no conflicts of interest.

## Supporting information


Appendix S1:

Appendix S2:

Appendix S3:

## Data Availability

The data that support the findings of this study are openly available in Dryad at: http://datadryad.org/stash/share/Okkn3f3UY78NW8pIt9yIEMBq9‐M0‐ouMo‐xW8KNjW78; https://doi.org/10.5061/dryad.8931zcs0r.
