# Supplementary material for: Rethinking Conservation and Restoration Strategies of Endangered and Key Medicinal Clavicarpa Plants in Yunnan‐Kweichow Plateau's Karst Areas Under Climate Change
Source: Ecol Evol. 2025 Jan 14;15(1):e70790. doi: 10.1002/ece3.70790 (PMC11732739; doi:10.1002/ece3.70790)
Supplement: Supplementary file 2 — Appendix S2: [file ECE3-15-e70790-s001.docx]

**Rethinking conservation and restoration strategies of endangered and key medicinal *Clavicarpa* plants in Yunnan-Kweichow Plateau's Karst areas under climate change**

Chao Luo^a,d,1^, Baiyang He^a,1^, Yulu Wu^a^, Yuteng Xue^a^, Huang Deng^a^, Shanman Li^a^, Xianghong Dong^b,c^* and Litang Lv^d^*

a College of Forestry, Guizhou University, Guiyang, 550025, China;

b Key Laboratory of Animal Genetics, Breeding and Reproduction in the Plateau Mountainous Region, Ministry of Education, Guizhou University, Guiyang, 550025, China;

c College of Animal Science, Guizhou University, Guiyang, 550025, China

d College of Life Science, Guizhou University, Guiyang, 550025, China

***Author for Correspondence**

* E-mail addresses: [*xhdong@gzu.edu.cn](mailto:*xmhhq2001@163.com) (XHD); * ltlv@gzu.edu.cn (LTL).

1 Chao Luo and Baiyang He contributed equally to this work and should be considered co-first authors.

**Supplementary data 2**

**MRI_ESM2_0:**

**Main results**

**Species distribution shifts**

The models projected varying degrees of range shifts for Clavicarpa species across different time periods under both SSP1-2.6 and SSP5-8.5 scenarios. The severity of range expansions for *I.tubulosa, I.apalophylla,* and *I.claviger* increased with higher SSP severity, while irregular changes in species ranges were observed for *I.pritzelii, I. wilsonii* and *I. guizhouensis* (Fig. S1; Table S4). Overall, under SSP1-2.6 and SSP5-8.5 scenarios, dramatic range expansions would occur for *I.tubulosa, I.apalophylla,* and *I.claviger* , ranging from 69.73% to 187.26% throughout the given time periods. Most of their habitat gains would occur in parts of East, Central, Southwest (mainly in Sichuan Basin), and Northwest (mainly in Tarim basin) China, which were far away from their current ranges. Additionally, the range for *I.pritzelii, I. wilsonii* and *I. guizhouensis* would change slightly and irregularly, while that of *. wilsonii* and *I. guizhouensis* would expand marginally, with gains ranging from -2.31% to -14.09%. Furthermore, except a northward shift in distribution centroid observed for *I.pritzelii,* there would be apparent southward shifts in distribution centroids for the other five species from current to the 2070s (Table S1). Notably, more or fewer areas of transitory fluctuations in the species ranges for all six species were observed throughout the entire time period (Fig. S1).

**Table S4.** Changes of distribution centriod and suitable grid numbers across multiple global climate models, scenarios, and time periods

| Species | Scenarios | Time | Distribution centroid | | Change in suitable grid number (%) |
| --- | --- | --- | --- | --- | --- |
|  |  | periods | Longitude | Latitude |  |
| *I. apalophylla* | SSP1-2.6 | 2030s | 110.82 | 28.60 | 70.69 |
|  |  | 2050s | 110.91 | 28.65 | 9.12 |
|  |  | 2070s | 110.79 | 28.75 | -5.64 |
|  | SSP5-8.5 | 2030s | 110.90 | 28.62 | 69.73 |
|  |  | 2050s | 111.23 | 28.93 | 17.42 |
|  |  | 2070s | 111.39 | 29.33 | 9.21 |
| *I. claviger* | SSP1-2.6 | 2030s | 112.19 | 29.77 | 186.96 |
|  |  | 2050s | 112.20 | 29.63 | 9.93 |
|  |  | 2070s | 112.09 | 29.66 | -3.50 |
|  | SSP5-8.5 | 2030s | 112.16 | 29.86 | 187.26 |
|  |  | 2050s | 112.24 | 29.83 | 18.75 |
|  |  | 2070s | 112.21 | 29.97 | 14.46 |
| *I. guizhouensis* | SSP1-2.6 | 2030s | 110.60 | 29.24 | 9.68 |
|  |  | 2050s | 110.26 | 29.28 | -14.09 |
|  |  | 2070s | 110.35 | 29.59 | 0.43 |
|  | SSP5-8.5 | 2030s | 110.78 | 29.17 | 16.01 |
|  |  | 2050s | 110.76 | 29.35 | -12.09 |
|  |  | 2070s | 110.94 | 29.76 | -0.58 |
| *I. pritzelii* | SSP1-2.6 | 2030s | 111.38 | 30.26 | 41.25 |
|  |  | 2050s | 111.69 | 30.31 | 6.76 |
|  |  | 2070s | 111.61 | 30.55 | -8.38 |
|  | SSP5-8.5 | 2030s | 111.53 | 30.24 | 46.54 |
|  |  | 2050s | 111.76 | 30.04 | 18.05 |
|  |  | 2070s | 111.82 | 29.89 | 20.72 |
| *I. tubulosa* | SSP1-2.6 | 2030s | 112.35 | 26.81 | 47.16 |
|  |  | 2050s | 111.95 | 27.10 | 10.81 |
|  |  | 2070s | 111.92 | 27.22 | 3.13 |
|  | SSP5-8.5 | 2030s | 112.47 | 26.78 | 42.64 |
|  |  | 2050s | 111.64 | 27.17 | 22.15 |
|  |  | 2070s | 110.33 | 27.75 | 19.51 |
| *I. wilsonii* | SSP1-2.6 | 2030s | 108.96 | 28.81 | -2.31 |
|  |  | 2050s | 109.14 | 28.65 | 5.02 |
|  |  | 2070s | 109.02 | 28.71 | -7.88 |
|  | SSP5-8.5 | 2030s | 109.03 | 28.69 | 1.25 |
|  |  | 2050s | 109.10 | 28.63 | 2.60 |
|  |  | 2070s | 108.60 | 28.65 | -10.33 |

**
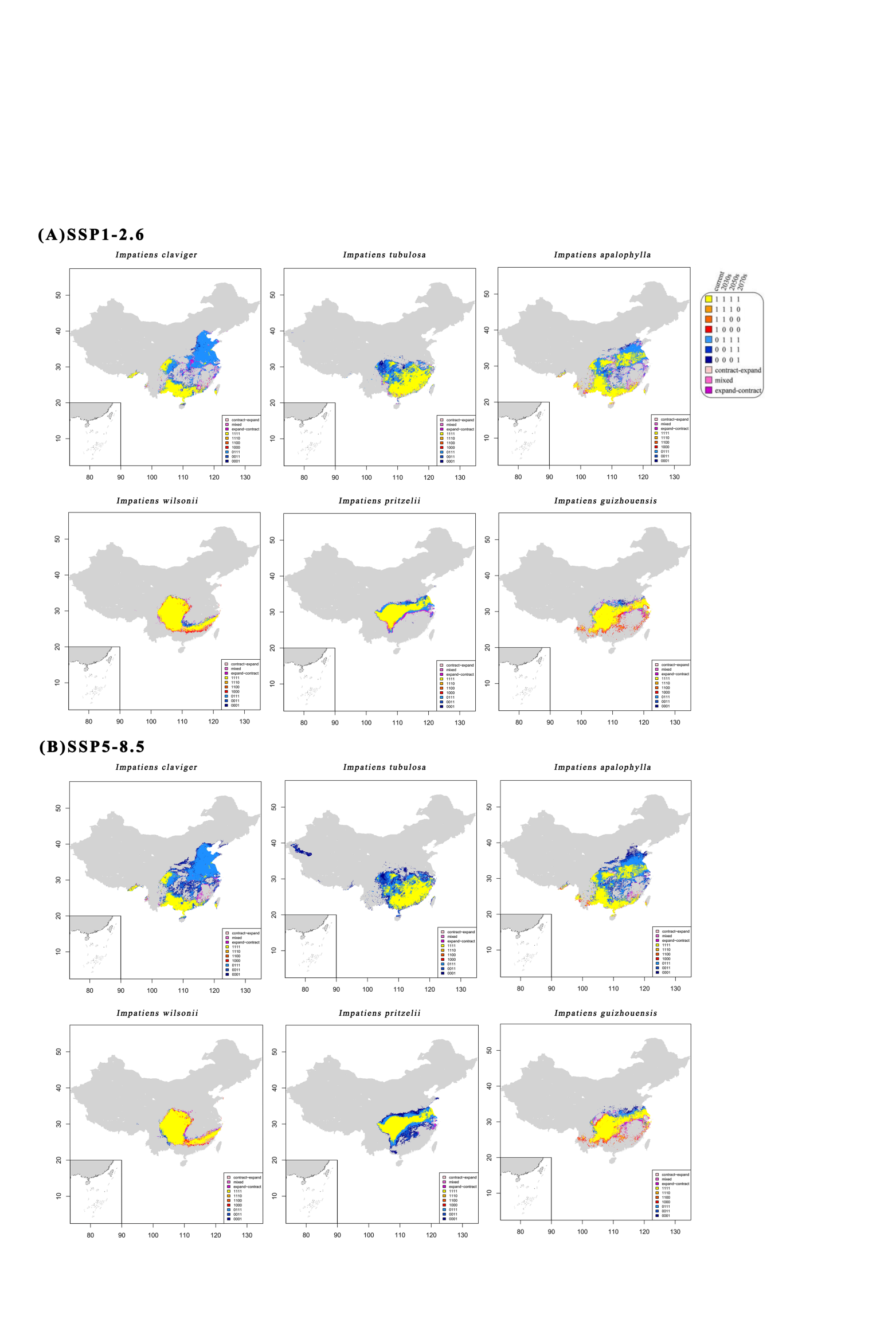
**

Fig. S1. Time-step maps detailing range shifts for the six species under two different Shared Socio-economic Pathways scenarios ((A): SSP1-2.6; (B): SSP5-8.5) and for four time periods (current, the 2030s, 2050s, and2070s). “1” and “0” in the legend indicate presence and absence of one species, respectively. The “contract-expand” group in the legend indicates “1001”, “1011”, and “1101”. The “mixed” group in the legend indicates “0101” and “1010”. The “expand-contract” group in the legend indicates “0100”, “0010”, and “0110”.

**Current and future species richness of the six species**

A significant increase in species richness of the species would predominantly occur in the south of the Yangtze River, including Sichuan Basin, the Yunnan-Guizhou Plateau, and the whole central and southern China across the given time periods under both SSP1-2.6 and SSP5-8.5 scenarios (Fig. S2). By the 2070s, species richness would be relatively high in Along the Yangtze River Basin, including the eastern region of Sichuan, Chongqing, Hunan, Hubei, Anhui, and the Yangtze River Delta region under these two scenarios.


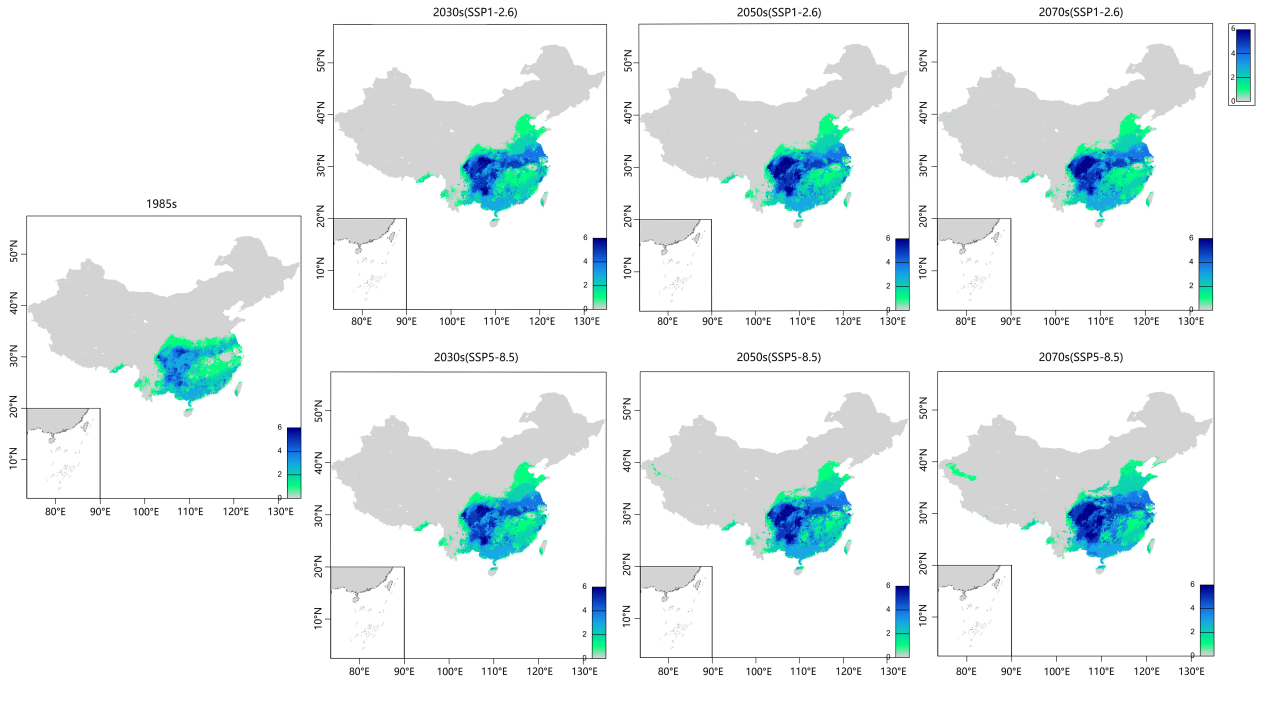


Fig. S2. Species richness maps cumulating the six species distributions forecasted under two different Shared Socio-economic Pathways scenarios (SSP1-2.6 and SSP5-8.5) and for four time periods (current, the 2030s, 2050s, and 2070s).

**EC-Earth3-Veg:**

**Main results**

**Species distribution shifts**

The models projected varying degrees of range shifts for Clavicarpa species across different time periods under both SSP1-2.6 and SSP5-8.5 scenarios. The severity of range expansions for *I.tubulosa, I.apalophylla, I.pritzelii,* and *I.claviger* increased with higher SSP severity, while irregular changes in species ranges were observed for *I. wilsonii* and *I. guizhouensis* (Fig. 3; Table 2). Under the SSP1-2.6 and SSP5-8.5 scenarios, significant expansions in the distribution range of *I.tubulosa, I. apalophylla,* and *I. claviger* are anticipated, with increases ranging from 32.13% to 143.62% over the specified time periods. Regarding *I.tubulosa, I. apalophylla,* and *I. claviger,* their habitats remained consistently suitable throughout the entire time period, primarily located in certain regions. Notably, only *I. apalophylla* and *I. claviger* exhibited a shared pattern of changes in their distribution, with significant increases in suitable habitats observed in various parts of Central, North China Plain, Sichuan Basin, and the Yangtze River Delta across different time periods (Table S5). In relation to *I. pritzelii* and *I. guizhouensis*, the majority of their consistently suitable habitats throughout the entire duration were primarily situated in Karst areas on the Guizhou Plateau, along the Yangtze River, and in the Yangtze River Delta region. Notably, more or fewer areas of transitory fluctuations in the species ranges for all six species were observed throughout the entire time period (Fig. S3)

**Table S5.** Changes of distribution centriod and suitable grid numbers across multiple global climate models, scenarios, and time periods

| Species | Scenarios | Time | Distribution centroid | | Change in suitable grid number (%) |
| --- | --- | --- | --- | --- | --- |
|  |  | periods | Longitude | Latitude |  |
| *I. apalophylla* | SSP1-2.6 | 2030s | 110.52 | 28.37 | 45.58 |
|  |  | 2050s | 110.87 | 28.88 | 9.45 |
|  |  | 2070s | 111.05 | 29.08 | 0.69 |
|  | SSP5-8.5 | 2030s | 110.54 | 28.46 | 39.02 |
|  |  | 2050s | 111.06 | 29.15 | 18.75 |
|  |  | 2070s | 111.55 | 30.19 | 12.46 |
| *I. claviger* | SSP1-2.6 | 2030s | 111.75 | 29.57 | 143.62 |
|  |  | 2050s | 111.94 | 30.01 | 8.84 |
|  |  | 2070s | 112.03 | 30.00 | 0.00 |
|  | SSP5-8.5 | 2030s | 111.68 | 29.73 | 136.75 |
|  |  | 2050s | 112.01 | 30.12 | 24.64 |
|  |  | 2070s | 112.17 | 30.57 | 17.98 |
| *I. guizhouensis* | SSP1-2.6 | 2030s | 110.53 | 29.28 | 13.25 |
|  |  | 2050s | 110.47 | 29.67 | -4.91 |
|  |  | 2070s | 110.67 | 29.81 | -5.73 |
|  | SSP5-8.5 | 2030s | 110.64 | 29.35 | 8.57 |
|  |  | 2050s | 110.54 | 29.61 | -4.86 |
|  |  | 2070s | 110.44 | 30.09 | -12.60 |
| *I. pritzelii* | SSP1-2.6 | 2030s | 111.42 | 30.65 | 21.52 |
|  |  | 2050s | 111.61 | 30.85 | 9.24 |
|  |  | 2070s | 111.63 | 30.75 | 7.43 |
|  | SSP5-8.5 | 2030s | 111.20 | 30.65 | 18.85 |
|  |  | 2050s | 111.75 | 30.70 | 20.56 |
|  |  | 2070s | 111.84 | 30.63 | 27.32 |
| *I. tubulosa* | SSP1-2.6 | 2030s | 112.86 | 26.84 | 40.17 |
|  |  | 2050s | 112.74 | 27.13 | 13.16 |
|  |  | 2070s | 112.74 | 27.19 | 0.40 |
|  | SSP5-8.5 | 2030s | 113.11 | 26.81 | 32.13 |
|  |  | 2050s | 112.80 | 27.10 | 15.97 |
|  |  | 2070s | 111.55 | 27.93 | 27.81 |
| *I. wilsonii* | SSP1-2.6 | 2030s | 109.34 | 29.12 | 4.94 |
|  |  | 2050s | 109.24 | 29.09 | -3.06 |
|  |  | 2070s | 108.99 | 29.28 | -9.13 |
|  | SSP5-8.5 | 2030s | 109.27 | 29.01 | 2.28 |
|  |  | 2050s | 109.50 | 29.53 | 9.78 |
|  |  | 2070s | 109.11 | 30.01 | -8.28 |

**
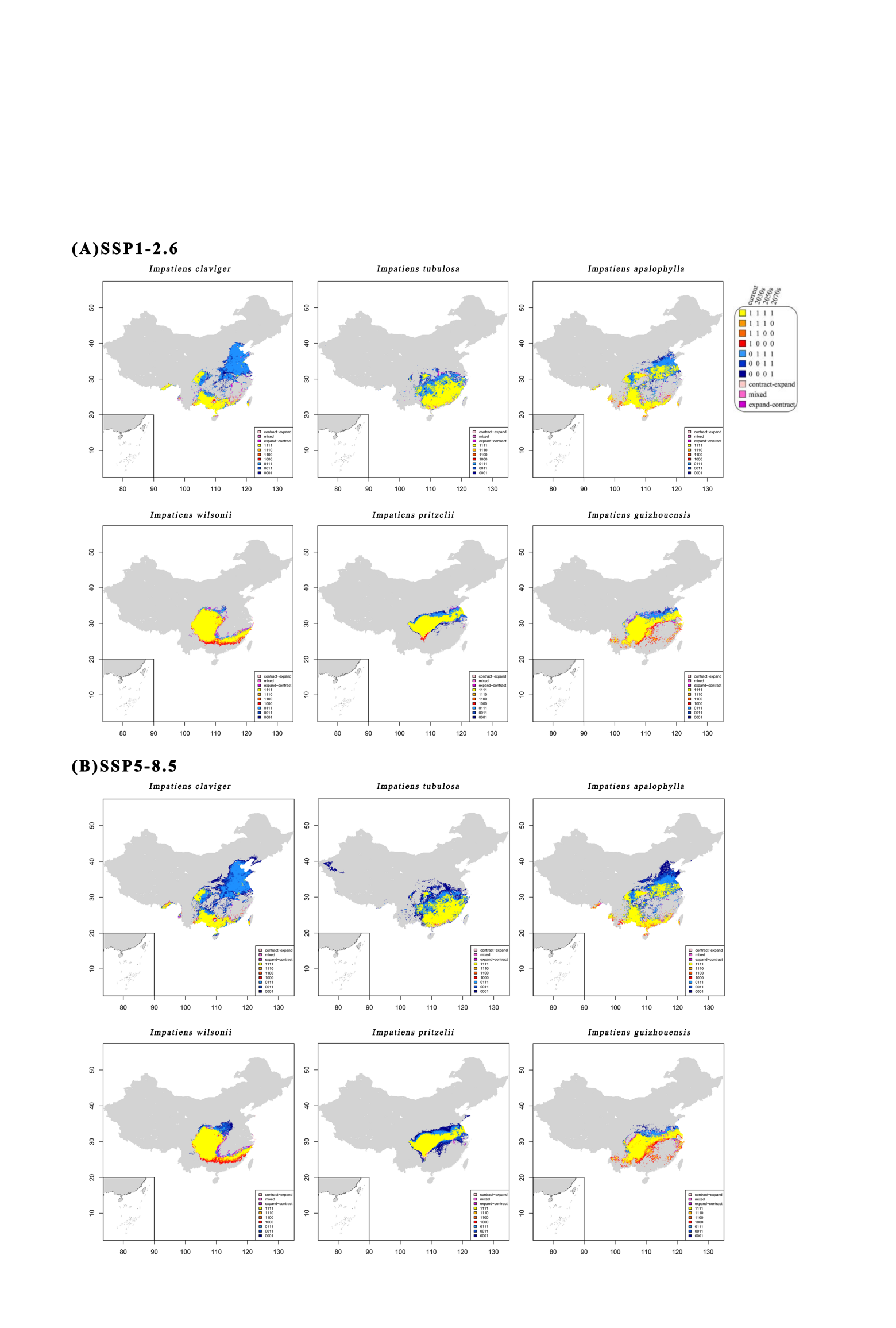
**

Fig. S3. Time-step maps detailing range shifts for the six species under two different Shared Socio-economic Pathways scenarios ((A): SSP1-2.6; (B): SSP5-8.5) and for four time periods (current, the 2030s, 2050s, and2070s). “1” and “0” in the legend indicate presence and absence of one species, respectively. The “contract-expand” group in the legend indicates “1001”, “1011”, and “1101”. The “mixed” group in the legend indicates “0101” and “1010”. The “expand-contract” group in the legend indicates “0100”, “0010”, and “0110”.

**Current and future species richness of the six species**

A significant increase in species richness of the six species would predominantly occur in Along the Yangtze River Basin, it includes humid areas such as Sichuan, Chongqing, Anhui, Hunan, Hubei, and Jiangxi across the given time periods under both SSP1-2.6 and SSP5-8.5 scenarios (Fig. S4). By the 2070s, species richness would be relatively high in Regions with tributaries of the Yangtze River, such as Sichuan, Chongqing, Hunan, Hubei, Anhui, Jiangxi, Shanghai, etc., especially near the Yangtze River basin under these two scenarios.


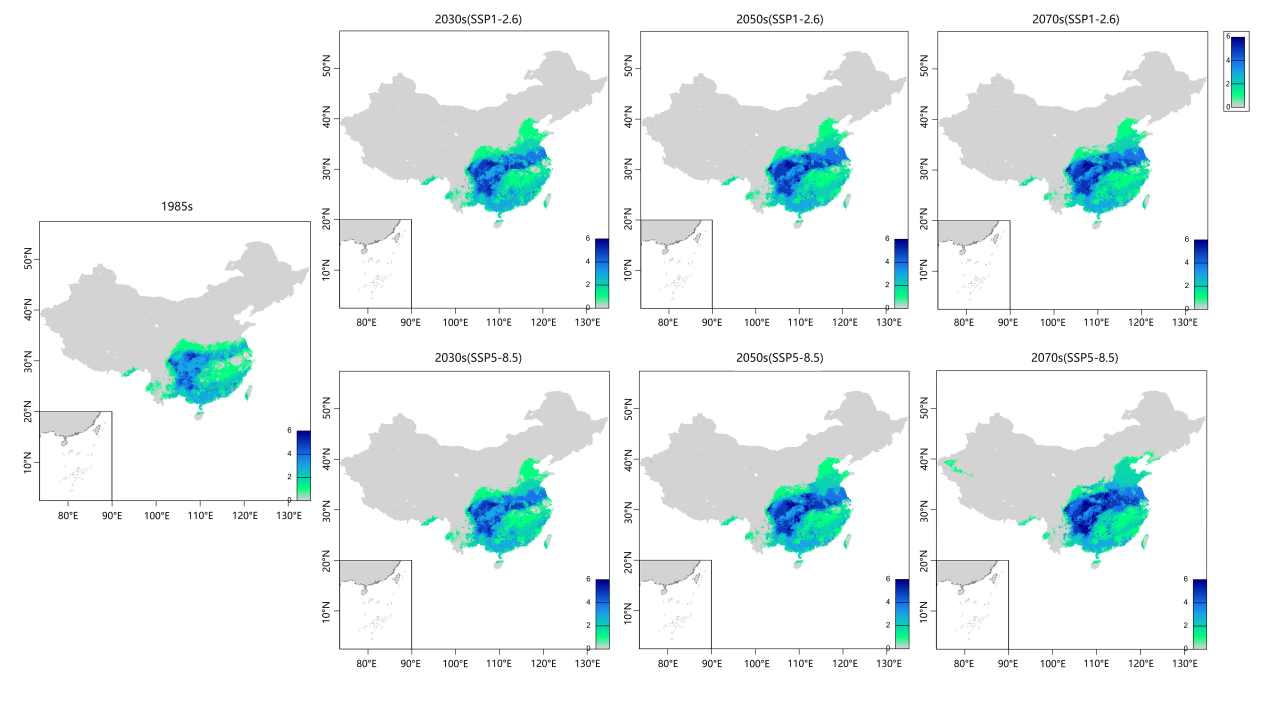


Fig. S4. Species richness maps cumulating the six species distributions forecasted under two different Shared Socio-economic Pathways scenarios (SSP1-2.6 and SSP5-8.5) and for four time periods (current, the 2030s, 2050s, and 2070s).
