# Supplementary material for: Rethinking Conservation and Restoration Strategies of Endangered and Key Medicinal Clavicarpa Plants in Yunnan‐Kweichow Plateau's Karst Areas Under Climate Change
Source: Ecol Evol. 2025 Jan 14;15(1):e70790. doi: 10.1002/ece3.70790 (PMC11732739; doi:10.1002/ece3.70790)
Supplement: Supplementary file 3 — Appendix S3: [file ECE3-15-e70790-s003.docx]

**Rethinking conservation and restoration strategies of endangered and key medicinal Clavicarpa plants in Yunnan-Kweichow Plateau's Karst areas under climate change**

Chao Luo^a,d,1^, Baiyang He^a,1^, Yulu Wu^a^, Yuteng Xue^a^, Huang Deng^a^, Shanman Li^a^, Xianghong Dong^b,c^* and Litang Lv^d^*

a College of Forestry, Guizhou University, Guiyang, 550025, China;

b Key Laboratory of Animal Genetics, Breeding and Reproduction in the Plateau Mountainous Region, Ministry of Education, Guizhou University, Guiyang, 550025, China;

c College of Animal Science, Guizhou University, Guiyang, 550025, China

d College of Life Science, Guizhou University, Guiyang, 550025, China

***Author for Correspondence**

* E-mail addresses: [*xhdong@gzu.edu.cn](mailto:*xmhhq2001@163.com) (XHD); * ltlv@gzu.edu.cn (LTL).

1 Chao Luo and Baiyang He contributed equally to this work and should be considered co-first authors.

**Table S1.** Detail information about the variables used in the present study.

| Type | Code | Description | Units |
| --- | --- | --- | --- |
| Bioclimatic factors | Bio1 | Annual mean temperature | ℃ |
|  | Bio2 | Mean monthly temperature difference | ℃ |
|  | Bio3 | Isothermality (Bio2/Bio7) (*100) | – |
|  | Bio4 | Temperature Seasonality (standard deviation*100) | – |
|  | Bio5 | Max temperature of warmest month | ℃ |
|  | Bio6 | Min temperature of coldest month | ℃ |
|  | Bio7 | Temperature annual range (Bio5-Bio6) | ℃ |
|  | Bio8 | Mean temperature of wettest quarter | ℃ |
|  | Bio9 | Mean temperature of driest quarter | ℃ |
|  | Bio10 | Mean temperature of warmest quarter | ℃ |
|  | Bio11 | Mean temperature of coldest quarter | ℃ |
|  | Bio12 | Annual precipitation | mm |
|  | Bio13 | Precipitation of wettest month | mm |
|  | Bio14 | Precipitation of driest month | mm |
|  | Bio15 | Variation coefficient of precipitation | – |
|  | Bio16 | Precipitation of wettest quarter | mm |
|  | Bio17 | Precipitation of driest quarter | mm |
|  | Bio18 | Precipitation of warmest quarter | mm |
|  | Bio19 | Precipitation of coldest quarter | mm |
| Topographic factors | Elev | Elevation | m |
| non-bioclimatic factors | Population | current and future population density | ind/km^2^ |

**Table S2**  Two scenarios of future shared socioeconomic

| **Scenario** | **Brief descriptions** |
| --- | --- |
| SSP1–2.6 | This scenario combines low social vulnerability and a low level of radiative forcing to achieve sustainable development and millennium development goals. In this scenario, many land use changes (especially the increase of global forest cover) and the dependence on resources and fossil energy are reduced. Finally, global warming is controlled in the range of 2 °C, and the radiation forcing is stable at 2.6 w/m^2^ in 2100. |
| SSP5–8.5 | This scenario involves a high level of radiation forcing, in which the traditional economy would be the guide for developing an energy-intensive fossil economy. Finally, the emissions of greenhouse gases are large, and the radiation forcing is stabilized at 8.5 w/m^2^ in 2100. |

**Table S3**  Mean model performance or generalization ability (n = 10, i.e. 10 repetitions) indicating by the area under the receiver operating characteristic curve (AUC).

| **species** | **AUC scores** |
| --- | --- |
| *I. claviger* | 0.928 |
| *I.guizhouensis* | 0.877 |
| *I.tubulosa* | 0.899 |
| *I.apalophylla* | 0.876 |
| *I.wilsonii* | 0.882 |
| *I. pritzelii* | 0.897 |

**Table S4**  The contribution rate of each environmental variable and cumulative contribution rate.

| *I. apalophylla* | | |  | *I. claviger* | | |  | *I. guizhouensis* | | |
| --- | --- | --- | --- | --- | --- | --- | --- | --- | --- | --- |
| Variables | Percent | Permutation |  | Variables | Percent | Permutation |  | Variables | Percent | Permutation |
|  | contribution | importance |  |  | contribution | importance |  |  | contribution | importance |
|  | rate (%) | rate (%) |  |  | rate (%) | rate (%) |  |  | rate (%) | rate (%) |
| Population | 23.1 | 12.1 |  | bio 18 | 57.4 | 3.5 |  | bio 14 | 34 | 17.2 |
| bio 12 | 22.6 | 11.5 |  | bio 08 | 8.4 | 5 |  | Population | 31.7 | 10.6 |
| bio 18 | 12.8 | 0.8 |  | bio 09 | 7.4 | 13.6 |  | Elev | 8.9 | 5.6 |
| bio 02 | 7.3 | 5.3 |  | Population | 6.4 | 4.8 |  | bio 09 | 8.9 | 10.9 |
| bio 19 | 6.4 | 10.4 |  | bio 02 | 4.6 | 22.7 |  | bio 06 | 6.4 | 47.8 |
| bio 03 | 6.3 | 4.5 |  | bio 03 | 4.2 | 8.2 |  | bio 19 | 5.4 | 1 |
| bio 17 | 5 | 2.6 |  | bio 15 | 3.4 | 6.7 |  | bio 13 | 3.3 | 4.7 |
| bio 08 | 3.7 | 3.6 |  | bio 04 | 3.1 | 0.9 |  | bio 12 | 0.7 | 1.1 |
| bio 04 | 3 | 2.1 |  | bio 04 | 1.4 | 22.1 |  | bio 04 | 0.3 | 0 |
| bio 15 | 2.5 | 0.7 |  | bio 17 | 1.4 | 1.9 |  | bio 08 | 0.2 | 1.1 |
| bio 06 | 2.4 | 31.1 |  | Elev | 0.5 | 0.2 |  | bio 16 | 0.1 | 0 |
| Elev | 1.1 | 1.9 |  | bio 01 | 0.5 | 0.7 |  | bio 01 | 0.1 | 0 |
| bio 09 | 1.1 | 7.6 |  | bio 11 | 0.4 | 6.5 |  |  |  |  |
| bio 11 | 1 | 1.6 |  | bio 10 | 0.4 | 0.1 |  |  |  |  |
| bio 01 | 0.7 | 1.2 |  | bio 19 | 0.3 | 0.8 |  |  |  |  |
| bio 10 | 0.4 | 0.6 |  | bio 07 | 0.2 | 2.4 |  |  |  |  |
| bio 07 | 0.4 | 1.3 |  | bio 14 | 0.1 | 0.1 |  |  |  |  |
| bio 14 | 0.1 | 1.1 |  |  |  |  |  |  |  |  |

|  | *I. pritzelii* |  |  | *I. tubulosa* | | |  | *I. wilsonii* | | |
| --- | --- | --- | --- | --- | --- | --- | --- | --- | --- | --- |
| Variables | Percent | Permutation |  | Variables | Percent | Permutation |  | Variables | Percent | Permutation |
|  | contribution | importance |  |  | contribution | importance |  |  | contribution | importance |
|  | rate (%) | rate (%) |  |  | rate (%) | rate (%) |  |  | rate (%) | rate (%) |
| bio 14 | 47.5 | 28.5 |  | bio 19 | 25.5 | 0.1 |  | Population | 24.5 | 10.7 |
| bio 19 | 16.9 | 21.7 |  | bio 14 | 20.4 | 0.7 |  | bio 04 | 24.1 | 18.9 |
| bio 04 | 16.5 | 11.5 |  | Population | 10.5 | 5.2 |  | bio 06 | 15.6 | 28.6 |
| bio 03 | 5.6 | 12.7 |  | bio 05 | 9.3 | 34 |  | bio 12 | 12.2 | 0 |
| bio 12 | 5 | 0.7 |  | bio 03 | 7.9 | 3.2 |  | bio 07 | 6.6 | 24 |
| Elev | 2.1 | 0.6 |  | bio 04 | 7.2 | 7.3 |  | bio 19 | 5.2 | 9.1 |
| bio 17 | 1.5 | 4.7 |  | bio 02 | 5.8 | 3.5 |  | bio 03 | 4.1 | 6.3 |
| bio 18 | 1.5 | 9.3 |  | bio 01 | 5.2 | 0.1 |  | Elev | 2.7 | 0 |
| bio 05 | 0.7 | 1.9 |  | bio 07 | 2.5 | 2.6 |  | bio 14 | 2.4 | 1.1 |
| bio 06 | 0.7 | 0.7 |  | bio 08 | 1.6 | 1.6 |  | bio 17 | 1.5 | 0 |
| bio 02 | 0.6 | 1.1 |  | bio 10 | 1.1 | 0 |  | bio 15 | 0.3 | 0.4 |
| bio 13 | 0.5 | 5.1 |  | bio 06 | 1.1 | 23.3 |  | bio 01 | 0.2 | 0 |
| Population | 0.5 | 0.8 |  | bio 16 | 0.8 | 2.1 |  | bio 10 | 0.2 | 0.5 |
| bio 08 | 0.3 | 0.1 |  | Elev | 0.5 | 13.1 |  | bio 11 | 0.1 | 0 |
| bio 11 | 0.2 | 0 |  | bio 09 | 0.2 | 0.2 |  | bio 08 | 0.1 | 0.1 |
| bio 09 | 0 | 0.5 |  | bio 15 | 0.1 | 1.5 |  | bio 02 | 0.1 | 0 |
| bio 16 | 0 | 0 |  | bio 12 | 0.1 | 1.6 |  |  |  |  |
| bio 07 | 0 | 0.1 |  |  |  |  |  |  |  |  |

Supplementary Table S5

Detail information about the bioclimatic variables used in the present study.

| **species** | **Brief descriptions** |
| --- | --- |
| *I. claviger* | 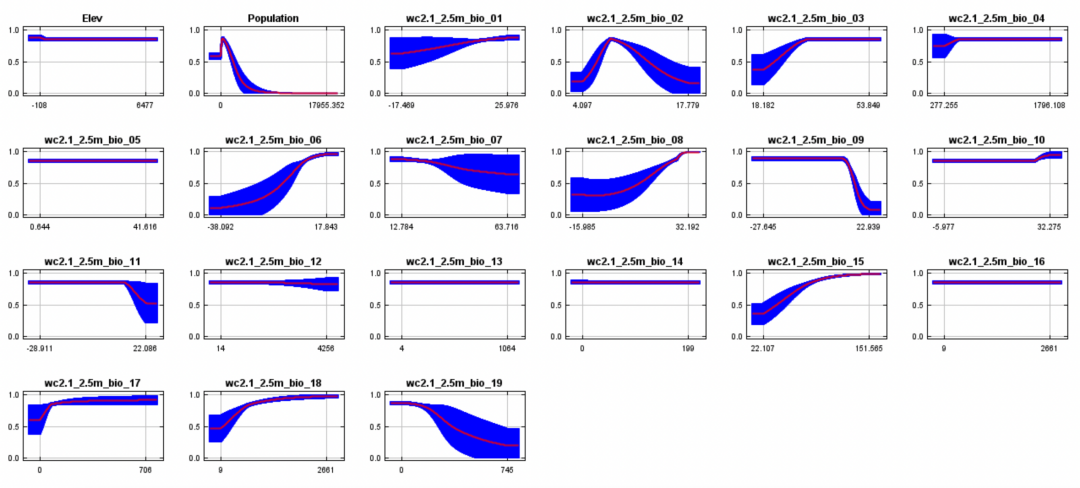 |
| *I.guizhouensis* | 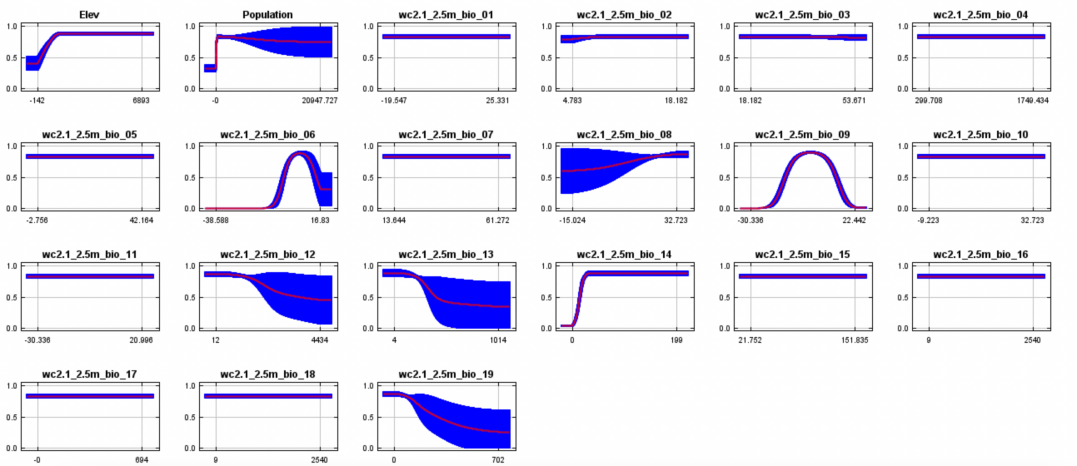 |
| *I.tubulosa* | 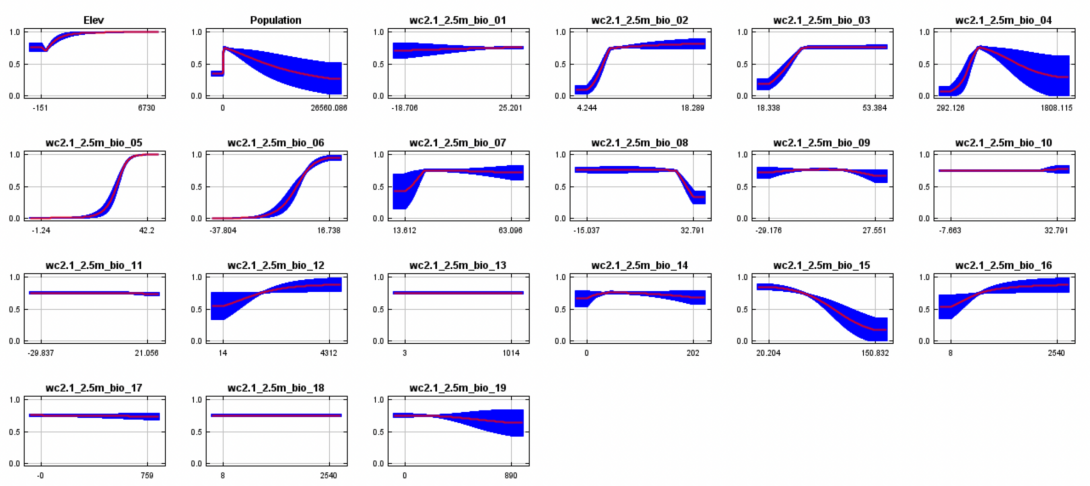 |
| *I.apalophylla* | 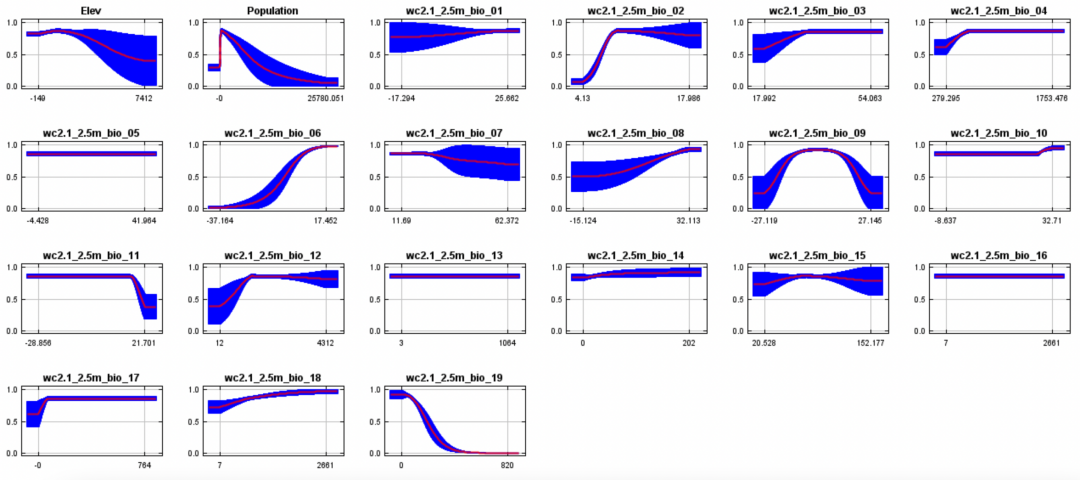 |
| *I.wilsonii* | 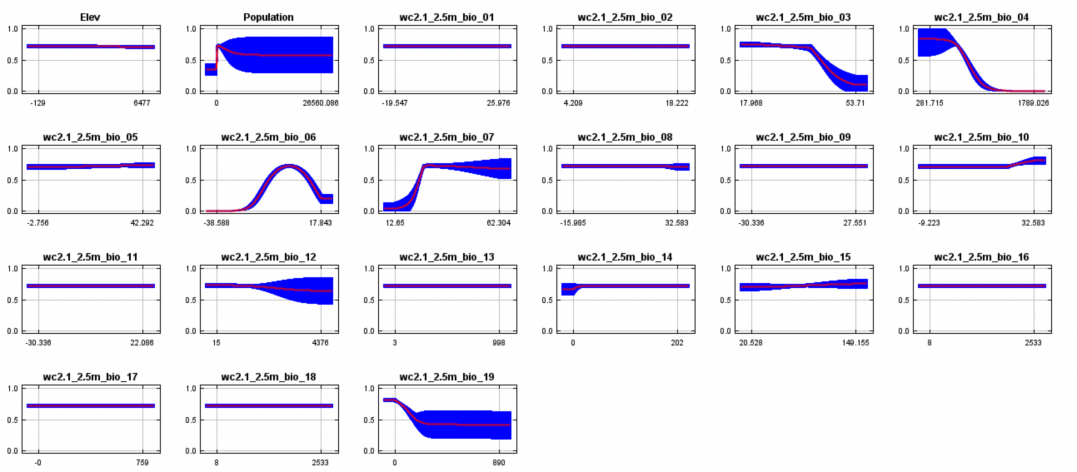 |
| *I. pritzelii* | 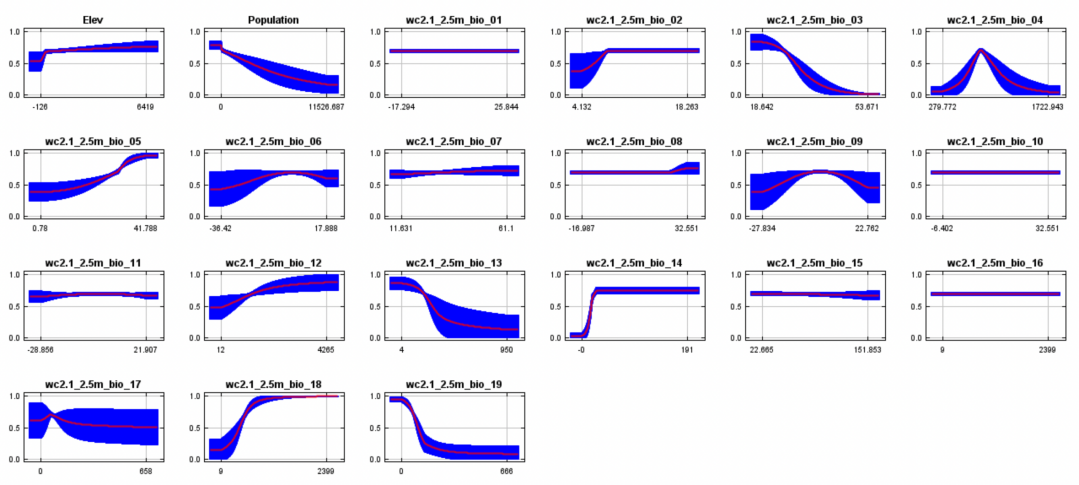 |
